# Testing for response shift in treatment evaluation of change in self‐reported psychopathology amongst secondary psychiatric care outpatients

**DOI:** 10.1002/mpr.1785

**Published:** 2019-06-17

**Authors:** Ingrid V.E. Carlier, Wessel A. van Eeden, Kim de Jong, Erik J. Giltay, Martijn S. van Noorden, Christina van der Feltz‐Cornelis, Frans G. Zitman, Henk Kelderman, Albert M. van Hemert

**Affiliations:** ^1^ Department of Psychiatry Leiden University Medical Centre Leiden The Netherlands; ^2^ Clinical Psychology Unit Institute of Psychology, Leiden University Leiden The Netherlands; ^3^ Department of Health Sciences, Hull York Medical School University of York Heslington UK; ^4^ Department of Methodology and Statistics Institute of Psychology, Leiden University Leiden The Netherlands

**Keywords:** longitudinal measurement invariance, mental health treatment, psychopathology, response shift, Symptom Questionnaire‐48 (SQ‐48)

## Abstract

**Objectives:**

If patients change their perspective due to treatment, this may alter the way they conceptualize, prioritize, or calibrate questionnaire items. These psychological changes, also called “response shifts,” may pose a threat to the measurement of therapeutic change in patients. Therefore, it is important to test the occurrence of response shift in patients across their treatment.

**Methods:**

This study focused on self‐reported psychological distress/psychopathology in a naturalistic sample of 206 psychiatric outpatients. Longitudinal measurement invariance tests were computed across treatment in order to detect response shifts.

**Results:**

Compared with before treatment, post‐treatment psychopathology scores showed an increase in model fit and factor loading, suggesting that symptoms became more coherently interrelated within their psychopathology domains. Reconceptualization (depression/mood) and reprioritization (somatic and cognitive problems) response shift types were found in several items. We found no recalibration response shift.

**Conclusion:**

This study provides further evidence that response shift can occur in adult psychiatric patients across their mental health treatment. Future research is needed to determine whether response shift implies an unwanted potential bias in treatment evaluation or a desired cognitive change intended by treatment.

## 1 INTRODUCTION

It is generally assumed that the subjective standard of measurement used in self‐report instruments is the same between time points and that comparisons made between them are valid measures of true change. However, there are indications that subjective standards of patients and their interpretations of, and response to, items may change across treatment. For instance, how patients view their symptoms may change due to recovery from the underlying mental disorder, improved cognitive abilities, and psychoeducation, which can affect how patients respond to self‐report items (e.g., Fokkema, Smits, Kelderman, & Cuijpers, 2013). This phenomenon is called a “response shift” (Golembiewski, Billingsley, & Yeager, 1976; Howard et al., 1979; Nolte, Mierke, Fischer, & Rose, 2016; Sprangers & Schwartz, 1999; Wu, 2016).

If persons change their perspective, this may alter the way they conceptualize, prioritize, and calibrate items. Consequently, three main types of response shifts have been identified: reconceptualization, reprioritization, recalibration (Nolte et al., 2016; Schwartz & Sprangers, 1999; Sprangers & Schwartz, 1999). *Reconceptualization* means that patients redefine the meaning of a concept such as “depression.” For instance, before treatment, patients had never considered somatic symptoms (e.g., sleep problems) as a component of their depression. However, after successful treatment, patients may consider somatic symptoms as part of their depression (Oort, 2005). *Reprioritization* means that the importance of specific symptoms changes in the overall measurement (Oort, 2005). For example, before treatment, when patients do not work due to sick leave, they may score items concerning concentration as not so important. However, after treatment, when patients resume their work, they may score these items as more important because they realize that concentration is crucial to their job performance. Finally, *recalibration* means a change in the patient's interpretation of response scale values. For example, after treatment, the Likert‐score of 1 (*rarely*) on the suicidal ideation item may represent another level of depression and rumination about suicide than before treatment. *Uniform recalibration* means a recalibration of the item scale, which influences all response options within an item and all subjects to the same extent and in the same direction. *Non‐uniform recalibration* means that the recalibration of the item scale differs in extent or direction across subjects and/or response options (Fokkema et al., 2013; Oort, 2005).

Although response shifts are not necessarily negative (as they represent adaptation), they may pose a threat to the measurement of change (Czobor & Volavka, 1996; Kaushal, 2016; Millsap, 2012; Nolte et al., 2016; Oort, 2005). For this reason, some authors prefer the term “response shift bias” instead of “response shift” (e.g., Fried et al., 2016; Kaushal, 2016; Shaw, Cross, & Zubrick, 2015; Tugwell & Knottnerus, 2014). Failing to account for response shift may result in overreporting or underreporting of true change. Response shift has been raised as a possible explanation for null or negative effects of some intervention programmes (e.g., Nixon & Werner, 2010; Smith, Schneider, Smith, & Ananiadou, 2004). Therefore, it is advised to consider testing for this potential source of bias (Barclay & Tate, 2014; Fokkema et al., 2013; Howard, Mattacola, Howell, & Lattermann, 2011; Ring, Höfer, Heuston, Harris, & O'Boyle, 2005; Shaw et al., 2015).

Several methods for testing/dealing with response shift have been proposed (e.g., Barclay‐Goddard, Epstein, & Mayo, 2009; Schwartz et al., 2013; Shaw et al., 2015). Two principally used methods are retrospective pretest (Hill & Betz, 2005; Howard, 1980; Howard & Dailey, 1979; Meyer, Richter, & Raspe, 2013; Nieuwkerk, Tollenaar, Oort, & Sprangers, 2007; Schwartz & Sprangers, 2010) and testing measurement invariance in confirmatory factor analyses (CFAs) or structural equation models (e.g., Millsap & Yun‐Tein, 2004; Oort, 2005; Vandenberg & Lance, 2000). The former would be appropriate in evaluation studies using a single‐item measure, and the latter, when a multi‐item measure is used (Shaw et al., 2015; e.g., present study).

Studies have already convincingly shown that response shift can occur across treatment of a chronic somatic disease (for an overview: e.g., Schwartz et al., 2006; Vanier, Leplège, Hardouin, Sébille, & Falissard, 2015). To date, only three studies specifically aimed at response shift testing regarding pretreatment versus post‐treatment self‐report scores amongst adult psychiatric patients (Fokkema et al., 2013; Nolte et al., 2016; Smith, Woodman, Harvey, & Battersby, 2016). All three studies revealed response shift, especially concerning depression items.

The purpose of this study is to contribute to research on response shift testing across mental health treatment in adult psychiatric patients. So far, no response shift studies were focused on a broad spectrum of psychopathological symptoms. Therefore, this study focused on psychopathology measured with the self‐report Symptom Questionnaire‐48 (SQ‐48) which was designed as public domain questionnaire in the context of Routine Outcome Monitoring (ROM; Carlier, Schulte‐Van Maaren, et al., 2012; Carlier et al., 2017). We tested the occurrence of all response shift types across treatment in a naturalistic sample of secondary psychiatric care outpatients. We expect above all response shift in the domain depression/mood, because it was suggested that depression in particular is sensitive to response shift (e.g., Nolte et al., 2016).

## 2 METHODS

### 2.1 Design and procedure

This study was conducted by the Department of Psychiatry of Leiden University Medical Centre (LUMC), using already available ROM data of a previous Dutch multicenter pre‐post treatment study (Carlier et al., 2017). Time between pre‐post assessments varied (Table 1), depending on how ROM was implemented (e.g., monthly, every 3–4 months, later). Consequently, the second assessment was not necessarily the end assessment (possibly interim assessment), nor was it due to meeting treatment goals or patient disengagement. For the purpose of this study, we selected ROM data of outpatients with common mental disorders who had both pre‐post treatment data of SQ‐48 (Carlier et al., 2017). General criteria to be eligible for ROM are: all psychiatric inpatients and outpatients, who are literate and have sufficient command of the Dutch language, and who are willing and able to complete self‐report instruments. Most common reason that patients are not eligible for ROM is insufficient command of the Dutch language, in which case they get treatment without ROM (de Beurs et al., 2011; instruments for non‐Dutch‐speaking patients in preparation). Within ROM, patients are enrolled in treatment (instead of research). Dropout or missing data in ROM generally have to do with patients who stop their treatment or no‐shows at their ROM measurement appointment, respectively. In the present study, we had no information regarding such data.

**Table 1:** Baseline characteristics of study sample (*N* = 206)

Female gender (%)		65.5
Age (years)		38.4 ± 12.1 (19–71)
Interval between pre/post (weeks)		18.9 ± 7.6 (4–42)
Ethnicity (%)		
	• Dutch	76.6
	• Other	5.8
	• Unknown	17.5
Psychopathology (%):		
	• Anxiety Disorders	36.4
	• Unipolar depressive disorders	39.8
	• Other	21.4
	• No diagnosis	2.4

*Note*. Data are expressed as percentages or means ± standard deviation, with range. Sample was used to test the factor invariance of the SQ‐48. Other psychopathology: somatoform disorders (most); personality disorders; bipolar disorders; disorders usually first diagnosed in infancy, childhood or adolescence; adjustment disorders; impulse‐control disorders not elsewhere classified; dissociative disorders; eating disorders; mental disorders due to a general medical condition not elsewhere classified. On the basis of Table S1 of Carlier et al. (2017) and adapted for this study.

Patients are administered a battery of measures which continues for as long as the patient is being treated. ROM measures generally may include: psychiatric interview (optional, Mini‐International Neuropsychiatric interview; Sheehan et al., 1998; Van Vliet & De Beurs, 2006), observer‐rated instruments (optional), and self‐report questionnaires (generic and disorder‐specific). Measures are administered by independent assessors (trained research nurses/psychologists) through computerized self‐report, which prevents missing data as item‐completion is necessary for progression to the next item. For a detailed description of Dutch ROM, see de Beurs et al. (2011). Dutch ROM is fairly comparable with ROM abroad (e.g., USA, UK) in terms of objectives; ROM data are collected systematically to assess treatment effectiveness in everyday clinical practice, to inform clinicians and patients about treatment progress (Carlier, Meuldijk, et al., 2012; Lambert, 2017; Lambert, Whipple, & Kleinstäuber, 2018). Also, implementation of ROM in clinical practice forms a common challenge in most countries (e.g., Boswell, Kraus, Miller, & Lambert, 2015; Essock, Olfson, & Hogan, 2015; Roe, Drake, & Slade, 2015). A possible difference with ROM abroad may be that Dutch ROM uses a less frequent but more comprehensive assessment battery (de Beurs et al., 2011).

Patients were treated in six comparable Dutch treatment centres (see Acknowledgements) of similar size and patient care organization. All patients were treated by psychiatrists or clinical psychologists/psychotherapists according to the principle of stepped‐care and (inter)national evidence‐based treatment guidelines (e.g., Cuijpers et al., 2013; Van Fenema, Van Der Wee, Bauer, Witte, & Zitman, 2012) concerning pharmacotherapy or psychotherapy (mainly cognitive behavioral therapy, CBT) or a combination of both (Van Fenema et al., 2012; Van Noorden, Van Fenema, van der Wee, Zitman, & Giltay, 2012). Treatment was not assigned, controlled, or influenced by the research team (Carlier et al., 2017).

The Medical Ethical Committee of the LUMC approved the general study protocol in which ROM is considered as an integral part of treatment process (no written informed consent is required). Patients may refuse ROM measurement and/or the anonymous use of their ROM data for scientific research without consequences (i.e., they receive necessary treatment). If patients refuse to take part in scientific research, their ROM data are removed from the ROM database (Carlier et al., 2017).

### 2.2 Participants

The study sample consisted of 206 outpatients (see Table 1).

Table 1 shows that there were 135 (65.5%) females and 71 (34.5%) males. Their age ranged from 19 to 71 years, with a mean of 38.4 years (*SD* = 12.1). Patients were mainly diagnosed with depressive and/or anxiety disorders (together 76.2%, Table 1). Other disorders mostly included somatoform disorders (about 14.3%, not in table). The mean interval between pretreatment and post‐treatment assessments was 18.9 weeks (*SD* = 7.6) (Table 1; Carlier et al., 2017).

### 2.3 Measure

#### 2.3.1 Symptom Questionnaire‐48 (SQ‐48)

The SQ‐48 is a generic self‐report questionnaire that assesses common psychopathological symptoms within seven subscales (seven factors with a total of 37 items): Aggression (four items), Mood/depression (six items), Somatic complaints (seven items), Anxiety (six items), Social phobia (five items), Agoraphobia (four items), Cognitive complaints (five items). Two additional subscales do not measure psychopathology and were therefore excluded for this study (Carlier et al., 2017): Vitality/optimism (six items), Work/study functioning (six items). All SQ‐48 items are for frequency on a 5‐point Likert scale (0: *Never*, 1: *Rarely*, 2: *Sometimes*, 3: *Often*, and 4: *Very often*). Mean administration time is 5.4 min (*SD =* 1.4; Carlier, Schulte‐Van Maaren, et al., 2012). The total score of the SQ‐48 in this study is based on the sum score of the seven psychopathology subscales (range from 0 to 148). A high total score indicates high levels of psychopathology/psychological distress.

CFA has been computed and demonstrated that the hypothesized factor structure of the SQ‐48 fitted well with the data in both a reference‐group of non‐patients (*n* = 516; comparative fit index [CFI] = 0.96; root mean square error of approximation (RMSEA) = 0.05) and a patient‐group with mainly depression and anxiety disorder (*n* = 242; CFI = 0.97; RMSEA = 0.06; Carlier, Schulte‐Van Maaren, et al., 2012). The SQ‐48 showed good internal consistency as well as good convergent and divergent validity in psychiatric outpatients and healthy reference‐group (Carlier, Schulte‐Van Maaren, et al., 2012). It also showed excellent test–retest reliability and good responsiveness to therapeutic change in psychiatric outpatients (Carlier et al., 2017). Detailed information about the development of the SQ‐48 is described elsewhere (see Carlier, Schulte‐Van Maaren, et al., 2012).

The Dutch SQ‐48 was translated into English according to evidence‐based guidelines for translation and cultural adaptations of questionnaires (Carlier, Schulte‐Van Maaren, et al., 2012; Wild et al., 2005; see Supporting information). Given that this study was Dutch, we have used the Dutch SQ‐48 version.

### 2.4 Statistical analyses

First, changes in total and sub‐scores of SQ‐48 were analysed using a doubly multivariate design with repeated measures in order to understand the impact of treatment and in preparation for response shift testing (Lix & Hinds, 2004). Cohen's *d* effect size was calculated. Because post‐treatment SD could be affected by treatment, we used baseline SD when computing Cohen's *d* (Cohen, 1992). Bear in mind that these effect sizes can only be interpreted when we find at least partial measurement invariance in most of the items (Byrne, Shavelson, & Muthén, 1989; Reise, Widaman, & Pugh, 1993; Steenkamp & Baumgartner, 1998; Vandenberg & Lance, 2000; see Appendix). Moreover, it may be affected by population heterogeneity (Greenland, Schlesaelman, & Criqui, 1986).

Second, we tested for response shift (longitudinal measurement invariance tests). Current statistical guidelines for response shift testing recommend using CFA (e.g., Elhai et al., 2013; Fokkema et al., 2013; Millsap & Hartog, 1988; Wu, 2016). We used weighted least squares means and variance (WLSMV) adjusted estimator (Beauducel & Herzberg, 2006; Hirschfeld & von Brachel, 2014), which takes the ordinal nature and non‐normal distribution of the data into account and resulted in the best model fit (e.g., Muthén & Asparouhov, 2002). Valid results were found in studies that also used WLSMV with similar sample sizes (e.g., Hukkelberg & Ogden, 2016; Nussbeck, Eid, & Lischetzke, 2006). In the original study of the SQ‐48 (Carlier, Schulte‐Van Maaren, et al., 2012), the factor structure was based on seven correlated psychopathology subscales with one layer, which is also used in this study (see Figure S1). Before computing longitudinal measurement invariance tests, we tested the model fit with the following fit indices (Hawes, Mulvey, Schubert, & Pardini, 2014): CFI (acceptable when CFI > 0.95; Hu & Bentler, 1999), Tucker‐Lewis Index (acceptable when TLI > 0.95; Hu & Bentler, 1999), and RMSEA (acceptable when RMSEA < 0.06; Hu & Bentler, 1999). Standardized mean difference (d) with 95% confidence interval (CI), adjusted for response shift, is reported (Oort, 2005; Del Re, 2013). CFAs were conducted using R‐package Lavaan (version 0.5.17 and 0.5.18; Rosseel et al., 2016).

More detailed information about CFA (including Models A, B, C, and D) and its interpretation in terms of response shift types can be found in the Appendix and Figure S1.

## 3 RESULTS

### 3.1 Change in severity of psychopathology

Table 2 summarizes tests of pretreatment and post‐treatment mean total scores (sum of subscale scores) and subscales.

**Table 2:** Comparison of SQ‐48 total and sub scores of pretreatment and post‐treatment data (*N* = 206)

		Pre‐treatment	Post‐treatment				
SQ‐48 sub scale	Items	Mean (*SD*)	Mean (*SD*)	Mean difference (*SE*)	*t*	*p*	d*
Aggression	4	5.1 (3.5)	4.6 (3.6)	0.5 (0.25)	1.86	.065	0.14
Agoraphobia	4	6.7 (4.2)	5.7 (4.4)	1.0 (0.23)	4.38	<.001	0.24
Anxiety	6	13.6 (5.5)	11.6 (6.3)	2.1 (0.35)	5.91	<.001	0.36
Cognitive complaints	5	9.8 (4.1)	8.4 (4.6)	1.4 (0.25)	5.71	<.001	0.34
Mood	6	12.2 (3.9)	10.8 (4.1)	1.4 (0.27)	5.14	<.001	0.36
Somatic complaints	7	10.2 (5.6)	8.5 (5.9)	1.7 (0.33)	5.28	<.001	0.30
Social phobia	5	9.0 (4.3)	7.6 (4.5)	1.4 (0.28)	4.85	<.001	0.33
SQ‐48 total score^a^	37	66.7 (21.5)	57.3 (25.8)	9.4 (1.46)	6.46	<.001	0.45

*Note*. Changes in total and sub scores are analysed using a doubly multivariate design with repeated measures. *p* values are two‐tailed. *SD* = standard deviation. *SE* = standard error. *t* = paired t‐test. d* = Cohen's *d* calculated with pretreatment *SD*. SQ‐48 = Symptom Questionnaire‐48.

a SQ‐48 total score composed of the following seven psychopathology sub scales: Mood, Anxiety, Somatic complaints, Social phobia, Agoraphobia, Aggression, and Cognitive complaints.

The total and subscores decreased at post‐treatment. The total score of the SQ‐48 at pretreatment ranged from seven to 121 with a mean of 66.66 (*SD =* 21.54), and at post‐treatment it ranged from four to 124 with a mean of 57.25 (*SD* = 25.84), which was a statistically significant decrease (psychological distress*treatment: *p* < .001; η2 = .193; *F* = 6.78; df = 7).

All subscale scores at pre‐ and post‐treatment showed significant decreases (all p‐values *<*0.001; Table 2 *)*, except for subscale Aggression. The Cohen's d effect size ranged from 0.14 to 0.45, which is considered small (Cohen, 1992).

### 3.2 Change in overall factor structure; reconceptualization

The 7‐factor psychopathology structure of the SQ‐48 (Figure S1) was analysed at both pretreatment and post‐treatment. The factor structure had a poor fit at pretreatment (not in table): CFI of 0.87, TLI of 0.86, RMSEA with a value of 0.143 (90% CI [0.139, 0.148]). The post‐treatment CFI of 0.92 and TLI of 0.91 indicate a better factor fit (not in table). The RMSEA of 0.183 (90% CI [0.179, 0.188]) indicate a worsening in factor fit. We computed a bootstrap analysis with 9,999 replicates and found that the difference in fit (ΔCFI) was significantly different from zero with the following 90% CI [−0.056, −0.007]. The configural model (Model A), an unconstrained model with pretreatment and post‐treatment combined resulted in a CFI of 0.90, TLI of 0.89, and RMSEA of 0.165 (90% CI [0.161, 0.168]). When looking at subscale‐specific fit indices, we see that the Mood subscale has a substantial lower CFI value (CFI = 0.78) compared with the other subscales (see Table 4). When looking at RMSEA, we found that four subscales had a poor fit: Mood, Anxiety, Somatic complaints, and Cognitive problems. Compared with the other fit indices, RMSEA is generally considered less reliable with relative modest sample sizes and large amount of parameters (Jackson, 2003).

All item‐specific pretreatment and post‐treatment factor loadings, threshold estimates for the four item categories, and residuals (residuals of pretreatment fixed to 1 with theta parametrization, see Appendix) of the configural model are demonstrated in Table 3. At pretreatment, all factor loadings were significant with *p* values of <.001 with the exception of Item 13 (“I considered my death or suicide,” *p* = .46), Item 19 (“I did not want to live anymore,” *p* = .05), Item 38 (“I felt hopeless,” *p* = .48), and Item 43 (“I wanted to hit people if I was provoked,” *p* = .22; see Table 3). These items, all within the Mood and Aggression subscale, loaded in the opposite direction (negative). At post‐treatment, we again saw that these items loaded negatively, but only Item 43 (Aggression) was not statistically significant (*p* = .16). We found that the factor loading of all items increased with the exceptions of Item 10 (“I argued with others”), Item 1 (“I was short of breath with minimal effort”), Item 25 (“I did not dare to go alone to a crowded shop”), and Item 36 (“I felt uncomfortable when other people looked at me”).

**Table 3:** Standardized parameter estimates for the configural invariance model of the longitudinal analysis

		Loadings		T1				T2				Residuals	
Factor	Item	T1	T2	Threshold 1	Threshold 2	Threshold 3	Threshold 4	Threshold 1	Threshold 2	Threshold 3	Threshold 4	T1	T2
Agression	10	0.76 (0.07)	0.71 (0.06)	−0.47 (0.09)	−0.01 (0.09)	0.67 (0.10)	1.19 (0.11)	−0.33 (0.09)	0.21 (0.09)	0.77 (0.10)	1.33 (0.12)	‐	0.47 (0.06)
	16	0.89 (0.06)	>0.99 (0.07)	−0.28 (0.09)	0.26 (0.09)	0.73 (0.10)	1.42 (0.13)	−0.04 (0.09)	0.45 (0.09)	1.03 (0.11)	1.71 (0.15)	‐	0.08 (0.09)
	21	0.58 (0.07)	0.59 (0.07)	−0.12 (0.09)	0.37 (0.09)	1.06 (0.11)	1.66 (0.15)	0.04 (0.09)	0.51 (0.09)	1.20 (0.11)	1.66 (0.15)	‐	0.65 (0.06)
	43	−0.13 (0.10)	−0.16 (0.11)	−0.41 (0.09)	0.37 (0.09)	1.24 (0.12)	2.34 (0.26)	−0.45 (0.09)	0.26 (0.09)	1.03 (0.11)	1.61 (0.14)	‐	0.98 (0.02)
Agoraphobia	4	0.83 (0.04)	0.90 (0.03)	−1.19 (0.11)	−0.76 (0.10)	0.05 (0.09)	1.24 (0.12)	−0.94 (0.10)	−0.43 (0.09)	0.39 (0.09)	1.22 (0.12)	‐	0.24 (0.04)
	8	0.73 (0.04)	0.83 (0.03)	−0.49 (0.09)	−0.13 (0.09)	0.34 (0.09)	1.22 (0.12)	−0.30 (0.09)	0.15 (0.09)	0.68 (0.10)	1.36 (0.12)	‐	0.36 (0.04)
	14	0.67 (0.06)	0.79 (0.05)	0.04 (0.09)	0.45 (0.09)	1.08 (0.11)	1.61 (0.14)	0.20 (0.09)	0.68 (0.10)	1.08 (0.11)	1.46 (0.13)	‐	0.45 (0.05)
	25	0.86 (0.04)	0.84 (0.03)	−0.48 (0.09)	−0.17 (0.09)	0.39 (0.09)	1.10 (0.11)	−0.39 (0.09)	0.06 (0.09)	0.70 (0.10)	1.19 (0.11)	‐	0.30 (0.04)
Anxiety	24	0.69 (0.04)	0.74 (0.04)	−0.94 (0.10)	−0.49 (0.09)	0.35 (0.09)	1.17 (0.11)	−0.86 (0.10)	−0.17 (0.09)	0.65 (0.10)	1.24 (0.12)	‐	0.48 (0.04)
	28	0.81 (0.03)	0.84 (0.02)	−0.55 (0.09)	−0.18 (0.09)	0.51 (0.09)	1.39 (0.13)	−0.43 (0.09)	0.06 (0.09)	0.59 (0.09)	1.36 (0.12)	‐	0.32 (0.03)
	33	0.75 (0.03)	0.83 (0.02)	−1.14 (0.11)	−0.65 (0.10)	0.07 (0.09)	1.14 (0.11)	−0.75 (0.10)	−0.35 (0.09)	0.41 (0.09)	1.22 (0.12)	‐	0.35 (0.03)
	41	0.83 (0.03)	0.89 (0.02)	−1.61 (0.14)	−1.06 (0.11)	−0.30 (0.09)	0.73 (0.10)	−1.19 (0.11)	−0.51 (0.09)	0.25 (0.09)	1.06 (0.11)	‐	0.25 (0.03)
	46	0.82 (0.03)	0.89 (0.02)	−1.10 (0.11)	−0.75 (0.10)	0.04 (0.09)	0.96 (0.10)	−0.86 (0.10)	−0.31 (0.09)	0.30 (0.09)	1.12 (0.11)	‐	0.25 (0.03)
	48	0.76 (0.03)	0.83 (0.03)	−1.89 (0.18)	−1.24 (0.12)	−0.48 (0.09)	0.56 (0.09)	−1.33 (0.12)	−0.85 (0.10)	−0.05 (0.09)	0.85 (0.10)	‐	0.36 (0.04)
Cognitive	2	0.53 (0.05)	0.65 (0.05)	−0.52 (0.09)	−0.15 (0.09)	0.59 (0.09)	1.83 (0.17)	−0.45 (0.09)	0.07 (0.09)	0.94 (0.10)	1.97 (0.19)	‐	0.64 (0.04)
problems	6	0.56 (0.05)	0.71 (0.04)	−0.75 (0.10)	−0.37 (0.09)	0.61 (0.09)	1.39 (0.13)	−0.62 (0.09)	−0.07 (0.09)	0.71 (0.10)	1.53 (0.14)	‐	0.57 (0.04)
	39	0.76 (0.04)	0.85 (0.03)	−1.19 (0.11)	−0.64 (0.09)	0.13 (0.09)	0.99 (0.11)	−0.86 (0.10)	−0.39 (0.09)	0.45 (0.09)	1.22 (0.12)	‐	0.34 (0.03)
	44	0.41 (0.07)	0.55 (0.05)	−0.32 (0.09)	0.02 (0.09)	0.47 (0.09)	1.39 (0.13)	−0.21 (0.09)	0.18 (0.09)	0.76 (0.10)	1.36 (0.12)	‐	0.75 (0.04)
	47	0.69 (0.04)	0.84 (0.03)	−1.83 (0.17)	−1.14 (0.11)	−0.30 (0.09)	0.68 (0.10)	−1.25 (0.12)	−0.73 (0.10)	0.16 (0.09)	1.06 (0.11)	‐	0.39 (0.04)
Mood	3	0.76 (0.04)	0.85 (0.04)	−1.17 (0.11)	−0.71 (0.10)	0.17 (0.09)	1.22 (0.12)	−0.96 (0.10)	−0.44 (0.09)	0.54 (0.09)	1.57 (0.14)	‐	0.38 (0.04)
	7	0.72 (0.04)	0.85 (0.04)	−0.49 (0.13)	−0.86 (0.10)	−0.04 (0.09)	0.94 (0.10)	−1.03 (0.11)	−0.47 (0.09)	0.39 (0.09)	1.33 (0.12)	‐	0.39 (0.04)
	13	−0.06 (0.07)	−0.46 (0.06)	−0.78 (0.10)	0.00 (0.09)	0.83 (0.10)	1.76 (0.16)	−0.58 (0.09)	−0.06 (0.09)	0.86 (0.10)	1.83 (0.17)	‐	0.93 (0.03)
	19	−0.14 (0.07)	−0.50 (0.06)	−0.94 (0.10)	−0.09 (0.09)	0.76 (0.10)	1.76 (0.16)	−0.73 (0.10)	−0.17 (0.09)	0.83 (0.10)	1.66 (0.15)	‐	0.91 (0.03)
	38	−0.06 (0.08)	−0.35 (0.06)	−1.39 (0.13)	−0.51 (0.09)	0.43 (0.09)	1.53 (0.14)	−1.17 (0.11)	−0.31 (0.09)	0.56 (0.09)	1.49 (0.13)	‐	0.96 (0.02)
	40	0.71 (0.04)	0.89 (0.04)	−1.39 (0.13)	−0.92 (0.10)	−0.05 (0.09)	0.96 (0.10)	−1.10 (0.10)	−0.59 (0.09)	0.32 (0.09)	1.19 (0.11)	‐	0.36 (0.04)
Somatic	1	0.72 (0.04)	0.69 (0.04)	−0.83 (0.10)	−0.22 (0.09)	0.58 (0.09)	1.24 (0.12)	−0.67 (0.10)	0.04 (0.09)	0.64 (0.09)	1.39 (0.13)	‐	0.53 (0.04)
complaints	5	0.77 (0.04)	0.89 (0.03)	−0.55 (0.09)	−0.13 (0.09)	0.65 (0.10)	1.14 (0.11)	−0.34 (0.09)	0.15 (0.09)	0.83 (0.10)	1.33 (0.12)	‐	0.30 (0.04)
	11	0.52 (0.06)	0.61 (0.05)	−0.55 (0.09)	0.01 (0.09)	0.96 (0.10)	1.89 (0.18)	−0.25 (0.09)	0.37 (0.09)	1.12 (0.11)	1.83 (0.17)	‐	0.67 (0.04)
	17	0.63 (0.05)	0.72 (0.04)	−0.52 (0.09)	−0.09 (0.09)	0.68 (0.10)	1.46 (0.13)	−0.32 (0.09)	0.12 (0.09)	0.92 (0.10)	1.27 (0.12)	‐	0.54 (0.04)
	22	0.55 (0.06)	0.68 (0.04)	−0.44 (0.09)	0.10 (0.09)	0.88 (0.10)	1.61 (0.14)	−0.30 (0.09)	0.28 (0.09)	0.97 (0.10)	1.66 (0.15)	‐	0.60 (0.04)
	26	0.67 (0.06)	0.82 (0.04)	−0.15 (0.09)	0.21 (0.09)	0.75 (0.10)	1.39 (0.13)	0.06 (0.09)	0.52 (0.09)	1.06 (0.11)	1.57 (0.14)	‐	0.43 (0.04)
	31	0.47 (0.07)	0.47 (0.07)	−0.07 (0.09)	0.35 (0.09)	0.85 (0.10)	1.39 (0.13)	0.17 (0.09)	0.56 (0.09)	1.17 (0.11)	1.89 (0.18)	‐	0.79 (0.04)
Socialphobia	23	0.62 (0.05)	0.76 (0.04)	−0.64 (0.09)	−0.18 (0.09)	0.75 (0.10)	1.66 (0.15)	−0.36 (0.09)	0.00 (0.09)	0.86 (0.10)	1.66 (0.15)	‐	0.49 (0.04)
	27	0.70 (0.05)	0.80 (0.03)	−0.47 (0.09)	0.00 (0.09)	0.59 (0.09)	1.39 (0.13)	−0.40 (0.09)	0.18 (0.09)	0.76 (0.10)	1.57 (0.14)	‐	0.42 (0.04)
	32	0.74 (0.04)	0.81 (0.03)	−0.61 (0.09)	−0.23 (0.09)	0.59 (0.09)	1.57 (0.14)	−0.47 (0.09)	0.04 (0.09)	0.85 (0.10)	1.97 (0.19)	‐	0.39 (0.04)
	36	0.57 (0.07)	0.47 (0.06)	−0.37 (0.09)	−0.15 (0.09)	0.45 (0.09)	1.03 (0.11)	−0.23 (0.09)	0.21 (0.09)	0.67 (0.10)	1.39 (0.13)	‐	0.75 (0.04)
	45	0.69 (0.05)	0.71 (0.05)	−1.46 (0.13)	−0.96 (0.10)	−0.21 (0.09)	0.83 (0.10)	−1.12 (0.11)	−0.61 (0.09)	0.20 (0.09)	1.06 (0.11)	‐	0.52 (0.05)

*Note*. T1 = Time 1. T2 = Time 2. Parenthetical values are standard errors.

The factor correlations of the factor model are presented in Table S1. All correlations were generally strong with a significance of *p* < .001, except for the correlations between Aggression and Agoraphobia (*p* = .189 at pretreatment and *p* = .008 at post‐treatment). Coefficients ranged from 0.099 (Aggression and Agoraphobia at pretreatment) till 0.902 (Cognitive problems and Anxiety at post‐treatment). Correlations increased at post‐treatment compared with pretreatment; exceptions were correlations between Mood and Cognitive problems, Mood and Anxiety, and Anxiety and Aggression.

The overall factor fit increased significantly over treatment and criteria for configural invariance could not be met. We found four items that loaded negatively on their common factor. Item 13 (“I considered my death or suicide”), Item 19 (“I did not want to live anymore”), and Item 38 (“I felt hopeless”) seemed to form a separate latent factor consisting of suicidal ideation and hopelessness. Subsequently, the model did not fit well within the Mood subscale which had consequences for the overall model fit. These negative factor loadings increased and were considered insignificant at pretreatment and significant at post‐treatment. Consequently, although items 13, 19, and 38 were already distinct from the rest of the mood items, an increase in factor loadings after treatment indicated a change in item scale meaning or *reconceptualization response shift*.

### 3.3 Factor metrics over time; reprioritization

In order to examine factor loading change in the factor model, the loadings were constrained between time points within the metric invariance model (Model B; Table 2). The change in factor fit was analysed (Table 4). The fit of the model remained similar with a CFI of 0.90 after constraining the factor loadings (Model B), with a significant decrease in chi‐square (Model A versus Model B; *p* = .002) but an insignificant decrease in CFI (*ΔCFI* 0.005). When looking at subscale specific *ΔCFI*, we found partial metric variance in factors Mood, Somatic complaints, and Cognitive problems (*ΔCFI <* 0.01; Model A versus B). When looking at item level, we found that item 26 within the Somatic subscale and item 47 within the Cognitive subscale caused these significant results. The metric variance detected in items 3, 7, and 40 was due to reconceptualization in the other Mood items, rather than reprioritization (see paragraph 3.2). Lifting constraints on these items resulted in invariant outcomes (Table 4, Table S2). Within these items, the factor loadings increased, suggesting *reprioritization response shift*.

**Table 4:** Comparison of pretreatment and post‐treatment partial equality constraints concerning SQ‐48 sub scales using partial measurement invariance procedures (*N* = 206).

Models	Fit indices	Mood	Anxiety	Somatic complaints	Socialphobia	Agoraphobia	Agression	Cognitive problems	All factors
Configural (Model A)	χ^2^	842.05	63.47	196.23	12.29	4.33	4.18	36.98	7969.70
	df	18	18	28	10	4	4	10	1216
	CFI	0.783	0.995	0.941	0.999	>0.999	>0.999	0.984	0.901
	RMSEA	0.473	0.111	0.171	0.033	0.020	0.015	0.115	0.165
Metric (Model B)	χ^2^	911.99	109.57	243.77	21.94	27.03	8.66	85.72	8372.70
	df	24	24	35	15	8	8	15	1253
	CFI	0.766	0.990	0.926	0.996	0.994	0.999	0.958	0.896
	RMSEA	0.425	0.132	0.171	0.048	0.108	0.020	0.152	0.166
Partial Metric (Model B)^a^	χ^2^	863.06	109.57	213.83	21.94	27.03	8.66	58.45	8331.85
	df	21	24	34	15	8	8	28	1248
	CFI	0.778	0.990	0.937	0.996	0.994	0.999	0.981	0.897
	RMSEA	0.442	0.132	0.161	0.048	0.108	0.020	0.080	0.166
Partial Strong (Model C)^a^	χ^2^	860.64	111.03	217.18	35.69	28.12	15.13	55.10	8259.03
	df	26	41	50	29	19	19	24	1332
	CFI	0.780	0.992	0.941	0.996	0.997	>0.999	0.981	0.899
	RMSEA	0.396	0.091	0.128	0.034	0.048	<0.001	0.080	0.159
Partial Strict (Model D)^a^	χ^2^	870.91	141.18	234.66	42.90	39.05	23.05	65.43	8424.66
	df	29	47	56	34	23	23	28	1364
	CFI	0.778	0.989	0.937	0.995	0.995	>0.999	0.978	0.897
	RMSEA	0.376	0.099	0.125	0.036	0.058	0.003	0.081	0.159
	χ^2^Diff	26.39	11.29	15.88	4.52	11.33	2.81	23.51	37.55
A vs. B	Δdf	6	6	7	5	4	4	5	37
	*p*	<.001	.008	.003	.255	.005	.431	<.001	.002
	ΔCFI	0.017	−0.005	0.014	0.003	0.006	0.001	0.026	0.005
	χ^2^Diff	14.29	11.29	7.12	4.52	11.33	2.81	12.82	35.24
A vs. B^a^	Δdf	3	6	6	5	4	4	3	32
	*p*	0.001	0.008	0.124	0.255	0.005	0.431	0.006	0.002
	ΔCFI	0.005	0.005	0.004	0.003	0.006	0.001	0.010	0.005
	χ^2^Diff	1.04	0.39	1.30	5.49	0.34	2.85	1.56	11.99
B^a^ vs. C^a^	Δdf	5	17	16	14	11	11	10	84
	*p*	>.999	.999	.999	.691	.996	.865	>.999	>.999
	ΔCFI	−0.002	−0.002	0.004	<0.001	−0.003	−0.001	0.002	0.002
	χ^2^Diff	13.21	12.40	11.89	6.66	8.60	8.02	10.87	31.70
C^a^ vs. D^a^	Δdf	3	6	6	5	4	4	4	32
	*p*	.002	.006	.024	.170	.025	.066	.019	.027
	ΔCFI	0.002	0.003	0.004	0.001	0.002	<0.001	0.004	0.002

*Note*. Analyses are conducted for all factors (sub scales) combined and separately for the sub scales. A = Configural model, no parameters constrained; B = Partial Metric model, factor loadings are constrained to be equal; Bᵃ = Partial Metric model, invariant factor loadings are constrained to be equal; Cᵇ = Partial Strong model, invariant variable thresholds and factor invariant loadings constrained to be equal; Dᶜ = Partial Strict model, invariant residual variances, invariant variable thresholds, and invariant factor loadings constrained to be equal. All constraints are computed with WLSMV estimation and theta parameterization. SQ‐48 = Symptom Questionnaire‐48. χ^2^ = Chi‐square test df = degrees of freedom for Ch‐square test. CFI = comparative fit index. χ^2^Diff = Chi‐square difference test. Δdf = degrees of freedom of Chi‐square difference test. *p* = *p* value. ΔCFI = delta comparative fit index.

a Lifting the equality constraints on loadings of items 3, 7, 26, 40, and 47

### 3.4 Thresholds over time; uniform recalibration

Thresholds are constrained between pretreatment and post‐treatment within the partial Strong invariance model (Model C; Table S3). Because Items 3, 7, 26, 40, and 47 were not invariant in the metric model, they were kept from further constraints in the strong model. Differences in thresholds between pretreatment and post‐treatment were analysed by testing the change in factor fit between Model B and Model C. The overall model fit remained the same (CFI = 0.90). The chi‐square difference tests per factor and for all factors combined were insignificant and ΔCFI did not exceed 0.01 (Model B versus C). No uniform change in measurement or *uniform recalibration response shift* could be detected.

### 3.5 Residual variances over time; non‐uniform recalibration

In order to test for change in residual variance between pretreatment and post‐treatment, residual variance was constrained between time points within the strict invariance model. Strict invariance assumes that the residual variance does not change during treatment. In order to assess partial strict invariance, residual variance is constrained between pretreatment and post‐treatment for all factors combined as well as each factor separately. Because strict measurement invariance was estimated with theta parameterization, which fixes residual variances to 1, item specific residuals could not be interpreted (Table S4). Because variance was found in the metric model, no constraints were conducted for Item 3, 7, 26, 40, and 47. The overall model fit remained similar (CFI *=* 0.90). Although the chi‐square difference test was significant for most factors, the *ΔCFI* did not exceeded the cutoff of <0.01 (Model C versus D; *p >* .05; *ΔCFI <* .01; Table 4), suggesting partial strict variance. This indicates that no shift in subjective standards of measurement or response scale values (*non‐uniform recalibration)* could be detected.

Finally, we analysed the standardized mean differences (d) between pretreatment and post‐treatment, adjusted for response shift. No significant decreases or increases in Cohen's *d* were found. Response shift effect sizes (95% CI) were: Aggression 0.25 (95% CI [0.05, 0.44]), Agoraphobia 0.25 (95% CI [0.05, 0.44]), Anxiety 0.36 (95% CI [0.16, 0.56]), Cognitive problems 0.32 (95% CI [0.13, 0.51]), Mood 0.37 (95% CI [0.07, 0.45]), Somatic complaints 0.29 (95% CI [0.10, 0.48]), and Social phobia 0.32 (95% CI [0.13, 0.52]). The final model parameters of the fully constrained model are presented in Table S4.

## 4 DISCUSSION

We tested the occurrence of response shift concerning self‐reported psychopathology in adult psychiatric outpatients across their mental health treatment. We found pretreatment and post‐treatment differences in factor structure and item factor loadings. In terms of response shift can be concluded that we found reconceptualization within the Mood subscale: items consisting of suicidal ideation and hopelessness became more distinct, and patients seemed to approach suicidal ideation after treatment as a separate concept from depression. So, it is possible that a considerable proportion of our sample may have less mood‐related symptoms after treatment without experiencing a decrease in suicidal ideations (see also Bringmann, Lemmens, Huibers, Borsboom, & Tuerlinckx, 2015; Nock, Hwang, Sampson, & Kessler, 2010). Second, we found reprioritization within at least two items of the subscales Somatic complaints and Cognitive problems. After treatment, patients seemed to place more value on these problems. Perhaps cognitive and somatic problems became more important when patients returned to work after sick leave. In conclusion, our hypothesis about response shift in especially the subscale depression/mood was only partly confirmed, as we also found response shift in other subscales. This may imply that not only depression is sensitive to response shift but also other psychopathology.

Our results are largely in line with current literature which indicates that response shift seems to be the rule rather than the exception. Only three studies were focused specifically on response shift in psychiatric patients (Fokkema et al., 2013; Nolte et al., 2016; Smith et al., 2016) and all found some level of response shift. Other relevant mental health studies without specific focus on response shift showed mixed results regarding its occurrence. Five studies in different psychiatric populations, using self‐report and/or clinician‐report instruments, found response shift indications: Barbosa‐Leiker, McPherson, Mamey, Burns, and Roll (2014); Czobor and Volavka (1996); Elhai et al. (2013); Fried et al. (2016); Boucekine et al. (2015). However, no response shift was found in the mental health studies by Quilty et al. (2013) with clinician‐rated scale and by de Beurs, Fokkema, de Groot, de Keijser, and Kerkhof (2015) with self‐report scale.

There is discussion on how strict the requirements should be concerning testing response shift by longitudinal measurement invariance (e.g., Fokkema et al., 2013). A first view states that full measurement invariance is an assumption that is too strict and, therefore, that comparisons of means across treatment are still meaningful when partial invariance is obtained and at least one item within each factor is invariant (Byrne et al., 1989; Steenkamp & Baumgartner, 1998). A second view is more strict and states that most (subscale) items should be invariant in order to make meaningful comparisons of the mean (Reise et al., 1993; Vandenberg & Lance, 2000; Wu, 2016). A third view assumes that true change in scores may be directly linked to respondents' changing perspective as a result of adaption, coping, or treatment (Boucekine et al., 2015; Oort, Visser, & Sprangers, 2009). In this view, response shift should not be considered as a measurement bias but as a true change.

Our study can be approached with all three views. Although we found response shift, this was present within a limited amount of items. This had no significant effect on the standardized mean difference between pretreatment and post‐treatment. Additionally, our patients were mainly treated with CBT, which can cause a shift in cognition and therefore may result in response shift. This is in line with response shift theory, which assumes that changes in a person's health status (e.g., diagnosis and treatment) are the requisite catalyst for response shift (Rapkin & Schwartz, 2004; Sprangers & Schwartz, 1999). This was confirmed by Wu who found response shift across treatment in depressed adolescents (Wu, 2015) but not in nonclinical adolescents (Wu, 2016). Accordingly, Ahmed, Sawatzky, Levesque, Ehrmann‐Feldman, and Schwartz (2014) found no response shift in chronic physically ill individuals with stable physical health, which supports the assumption that response shift is not expected in patients with relatively stable health conditions (Ahmed et al., 2014). Finally, there may also be other potential explanations for our results then response shift. One of these alternative explanations is a decrease of variability of items after treatment (Fried et al., 2016). Due to a decrease of severity, items may approach a mean of zero, resulting in small *SD*s that cannot exhibit substantial correlations anymore. This would explain variance of certain symptoms that may have low severity amongst a treated sample (e.g., acute suicidal ideation). However, in our study the *SD*s slightly increased and factor fit increased, suggesting that it is unlikely that this explains our findings.

A strength of this study is that ROM data were collected in a naturalistic sample of real‐life patients. We measured a wide range of psychological symptoms in a broad sample of adult psychiatric outpatients. Also, we have examined all response shift types, which has been done so far in only two adult mental health care studies (Fokkema et al., 2013; Nolte et al., 2016). This study also has limitations. We had no detailed individual information on therapists or types of treatments. So, for instance, it is not clear whether response shift varied by treatment type (psychotherapy and pharmacotherapy). Also, treatment length for participants varied, depending on the specific treatment required and its progress. It may be noted, however, that response shift already can occur after only 1 month of mental health treatment (e.g., Elhai et al., 2013; Latini et al., 2009). Moreover, after dividing our data in treatment longer—and shorter than 16 weeks (median), we found response shifts in the same items for both strata, with exception of Item 38 and 39 (Tables S5 and S6). Note that these sensitivity analyses should be interpret with caution because of limited sample sizes. Our sample size was also not large enough to examine potential subgroup differences regarding response shift between mental disorders. Finally, generalization of our results is at least limited to Dutch‐speaking patients. Generalization may also be limited by our study population (outpatients), our design (observational pre‐post‐treatment), and our instrument (generic self‐report questionnaire). However, this is unlikely because response shift has also been found concerning inpatients (e.g., Elhai et al., 2013; Nolte et al., 2016), randomized controlled trials (e.g., Fokkema et al., 2013), disease‐specific self‐report questionnaires (e.g. Elhai et al., 2013; Fokkema et al., 2013; Fried et al., 2016), and clinician‐rated instruments (e.g., Fried et al., 2016).

Future research with multiple follow‐ups could specify more exactly what type of response shift occurs at what moment across treatment (soon after the beginning of treatment or after a certain duration of it). Second, further research may evaluate the relative importance of the response shift types (Jakola, Solheim, Gulati, & Sagberg, 2016). For example, it was suggested that recalibration is the only true response shift, because reprioritization and reconceptualization can be seen as coping strategies instead of response shifts (Blome & Augustin, 2015; Gerlich et al., 2016). Third, more research is needed on predictors of which psychiatric patients may experience response shift (Daltroy, Larson, Eaton, Phillips, & Liang, 1999; Sprangers & Schwartz, 1999; Wu, 2016; Rapkin, Garcia, Michael, Zhang, & Schwartz, 2017). For instance, response shift seemed more likely to occur in psychotherapy patients than in those treated with medication (Fokkema et al., 2013; Fried et al., 2016; Uher et al., 2008). Additionally, further response shift research is needed to examine possible differences in mental disorders.

On the whole, this study provides additional evidence that response shift may occur in adult psychiatric patients across treatment. The exact meaning of this response shift is not clear: is it an unwanted potential bias in treatment evaluation or mainly a coping strategy and desired cognitive change intended by mental health treatment (e.g., CBT)? Future research in this area would be able to give more clarity on this question.

## DECLARATION OF INTEREST STATEMENT

The authors declare that there are no conflicts of interest in relation to the subject of this study.

## FUNDING SOURCES

This study was funded by the Dutch mental healthcare provider GGZ Rivierduinen.

## Supporting information

Table 1 Supplementary material
*Correlation matrix for latent factors in the configural invariance model*
Table 2 Supplementary material
*Standardized parameter estimates for the partial metric invariance model of the longitudinal analysis*
Table 3 Supplementary material
*Standardized parameter estimates for the partial strong invariance model of the longitudinal analysis*
Table 4 Supplementary material
*Parameter Estimates for the partial strict invariance model of the longitudinal analysis*
Table 5 treatment shorter than 16 weeks (median)
Comparison of pre‐ and post‐treatment partial equality constraints concerning SQ‐48 sub scales using partial measurement invariance procedures (*N* = 90)
Table 6 treatment longer than 16 weeks (median)
Comparison of pre‐ and post‐treatment partial equality constraints concerning SQ‐48 sub scales using partial measurement invariance procedures (*N* = 116)
Figure 1 Diagram of the SQ‐48 seven‐factor model, visualizing factor covariances, factors, item factor loadings, items and residuals, and item tresholds. Item subjects within qoutation marks. For the analyses described in this paper, the seven factor models of pre‐ and post‐treatment were hierarchically constrained to be equal to each other as follows:
Model A: no constraints
Model B: factor loadings constrained to be equal between pre‐ and post‐treatment
Model C: factor loadings and thresholds constrained to be equal between pre‐ and post‐treatment
Model D: factors loadings, thresholds, and variances constrained to be equal between pre‐ and post‐treatent

## APPENDIX 1

### THE USE OF CONFIRMATORY FACTOR ANALYSIS (CFA) IN THE CONTEXT OF RESPONSE SHIFT TESTING

A CFA framework allows for testing the structure of item sets and how they measure hypothesized latent variables. Pretreatment CFA can be compared with post‐treatment CFA frameworks in order to detect if response shifts occurred during treatment. In a series of nested CFA models (Models A, B, C, and D in the present study), hierarchal equality constraints on items' factor loadings, thresholds, and residual variances were applied. Model A contains a CFA model without any constraints between timepoints; Model B has loadings constrained to be equal between timepoints; Model C contains constrained loadings and thresholds; Model D contains constrained loadings, thresholds and residuals. A drop in model fit after constraint indicates inequality across time points (longitudinal measurement invariance tests; Fokkema et al., 2013; Millsap, 2012; Vandenberg & Lance, 2000; Widaman, Ferrer, & Conger, 2010). For example, when Model B has a substantial lower model fit compared with Model A, this suggests that loadings were substantially different between time‐points.

Equality was tested with chi‐square difference tests. However, as these tests are highly dependent on sample size, the more robust *ΔCFI* was calculated to see whether the CFI value was substantially different between CFA models (*ΔCFI* > 0.01;Gregorich, 2006; Kim, 2005 ; Rutkowski, 2013). A decrease in model fit is considered significant with a chi‐square difference test (*χ*) above *p =* .05, in conjunction with a *ΔCFI* above 0.01 (Chen, Curran, Bollen, Kirby, & Paxton, 2008; Cheung & Rensvold, 2002; Hu & Bentler, 1998). When full measurement invariance could not be obtained, the equality constraints of the non‐invariant parameters were lifted in order to assess partial measurement invariance (Oort, 2005; Wu, 2016).

These longitudinal measurement invariance tests were used as a framework to test the occurrence of four types of response shifts: reconceptualization, reprioritization, uniform recalibration, and non‐uniform recalibration.

#### Reconceptualization

A factor model is assumed to be configural invariant, meaning that the same factor loading pattern is present at both time points (pre/post). Each item should load on the same common factor, both at pretreatment and post‐treatment. When items load on different latent factors after treatment, this is indicative for a shift in concept. Violation of configural invariance is indicative for the occurrence of reconceptualization (Oort, 2005).

To test for configural invariance, the 7‐factor structure was fitted for both pretreatment and post‐treatment (Gregorich, 2006). The factor model is assumed to be configural invariant, when the difference of fit indices between pretreatment and post‐models is insignificant. We used bootstrapping (9,999 replicates) to test whether this difference is statistically significant (Canty, 2002; Oort, 2005). Furthermore, we compared the item‐specific factor loadings in order to check for salient changes of factor loading directions.

#### Reprioritization

The metric invariance model requires corresponding factor loadings to be equal across time points. An increase or decrease in factor loadings after treatment suggests that there is response shift in the form of reprioritization (Oort, 2005); for example, items seem to be more or less indicative for a certain latent factor. We computed a configural model with both time points (pretreatment and post‐treatment) combined, without equality constraints (Model A, see Table 4). We then computed a metric model with the factor loadings constrained equally between time points (Model B, see Table 4); if factor loadings between pretreatment and post‐treatment are similar, this should result in a similar model fit. A chi‐square difference test was conducted and a *ΔCFI* was calculated to measure whether the chi‐square and CFI significantly decreased after constraint (*χ = p <* .05; *ΔCFI > 0.01*). When the factor fit of Model B and Model A are similar, the result of the chi‐square difference test should be insignificant and the *ΔCFI* should be smaller than 0.01 (Gregorich, 2006; Kim, 2005; Rutkowski, 2013). Analyses were computed for the whole 7‐factor psychopathology model at once as well as per subscale separately. If invariance on subscale level was not met, we further examined partial item‐level measurement invariance (Wu, 2016).

#### Uniform recalibration

To test for uniform recalibration response shift, we must assess whether the regressions of items onto their associated common factors yield similar threshold values across time‐points. When equality is established, the model shows strong invariance, meaning that there is no indication for uniform recalibration (Oort, 2005). In other words, patients appraise the SQ‐48 item response options after treatment the same as before treatment. However, when there is variance, treatment may have changed a patient's idea of the amount of the hopelessness indicated by the answer option “often” (item 38; Oort, 2005). We computed a third model with both factor loadings (when invariant in the metric model) and thresholds constraint to be equal between time points (strong invariance model; Model C, see Table 4) and compared the factor fit with that of Model B. If threshold values are equal between pre‐ and post‐treatment, model fit should be similar after thresholds are constrained to be equal between time points. A chi‐square difference test in conjunction with *ΔCFI* was calculated in order to quantify the differentiations (χ = *p* > .05; ΔCFI <0.01; Gregorich, 2006; Rutkowski, 2013). Strong invariance tests were computed for the whole 7‐factor model at once, per subscale separately. When reprioritization or recalibration was found, the variant items were excluded from further constraints (partial measurement invariance; Wu, 2016).

#### Non‐uniform recalibration

When observed variance estimates across time‐points are compared, changes should reflect differences in common factor variation rather than contamination by changes in residual variation. Equality between pre‐ and post‐treatment residual variances is called strict invariance. Changes in residual variance assume the presence of non‐uniform recalibration response shift. Non‐uniform recalibration means that some SQ‐48 item response options are associated with a greater level of that item's specific construct and other response options are not. For example, the response option “sometimes” is related to greater levels of hopelessness (item 38) than the response option “rarely” (Oort, 2005). These non‐uniform recalibrations result in changes in variances that can not be attributed to change in common factor variances, i.e. residuals (Oort, 2005). In order to test the equality of residual variances, theta parameterization is used. Theta parameterization is presently the most reliable method for constraining residual variance with WLSMV estimation (Hirschfeld & von Brachel, 2014; Muthén & Asparouhov, 2002). The theta approach fixes the residual variance into 1 for all variables in the reference group (pre‐treatment). In the strict invariance model, the residuals of the post‐treatment group are also fixed into 1, in order to test the residual equality between pre‐ and post‐treatment (Muthén & Asparouhov, 2002). Equality constraints on corresponding factor loadings, thresholds, and residual variances were computed in the strict invariance model (Model D, see Table 4). If residual variances are equal between pre‐ and post‐treatment, Model D factor fit should be similar as Model C. The differences in factor fit of Model D in comparison with Model C were compared in order to detect discrepancies. Equality was assumed, when the chi‐square difference test was insignificant (*p* ≥ .05) and *ΔCFI <* 0.01 (Gregorich, 2006; Rutkowski, 2013). Analyses were computed for the whole 7‐factor model at once and per subscale separately. Items that were not invariant prior to the strict invariance model were not constrained. If invariance on subscale level in the strict model was not met, we further examined partial item‐level measurement invariance (Wu, 2016).
