# Supplementary material for: Testing for response shift in treatment evaluation of change in self‐reported psychopathology amongst secondary psychiatric care outpatients
Source: Int J Methods Psychiatr Res. 2019 Jun 17;28(3):e1785. doi: 10.1002/mpr.1785 (PMC6852603; doi:10.1002/mpr.1785)
Supplement: Supplementary file 1 — Table 1 Supplementary material Correlation matrix for latent factors in the configural invariance model Table 2 Supplementary material Standardized parameter estimates for the partial metric invariance model of the longitudinal analysis Table 3 Supplementary material Standardized parameter estimates for the partial strong invariance model of the longitudinal analysis Table 4 Supplementary material Parameter Estimates for the partial strict invariance model of the longitudinal analysis Table 5 treatment shorter than 16 weeks (median) Comparison of pre‐ and post‐treatment partial equality constraints concerning SQ‐48 sub scales using partial measurement invariance procedures (N = 90) Table 6 treatment longer than 16 weeks (median) Comparison of pre‐ and post‐treatment partial equality constraints concerning SQ‐48 sub scales using partial measurement invariance procedures (N = 116) Figure 1 Diagram of the SQ‐48 seven‐factor model, visualizing factor covariances, factors, item factor loadings, items and residuals, and item tresholds. Item subjects within qoutation marks. For the analyses described in this paper, the seven factor models of pre‐ and post‐treatment were hierarchically constrained to be equal to each other as follows: Model A: no constraints Model B: factor loadings constrained to be equal between pre‐ and post‐treatment Model C: factor loadings and thresholds constrained to be equal between pre‐ and post‐treatment Model D: factors loadings, thresholds, and variances constrained to be equal between pre‐ and post‐treatent [file MPR-28-e1785-s001.docx]

**Supporting Information**

**Testing for response shift in treatment evaluation of change in self-reported psychopathology amongst secondary psychiatric care outpatients**

**SQ-48**

| Name of the Patient:  Today’s Date:  Date of Birth: |
| --- |

**Instruction:**

Try to answer the following propositions fairly and accurately. There are no ‘right’ or ‘wrong’ answers. Give the answer that best expresses the number of times you have felt the following ways **last week, including today**. The answer which comes to your mind first is often the best answer.

Note: If you did not work or study or have not been able to do so, then you can skip the questions 9, 15, 20, 30 and 35.

| How much trouble did you have: | | never  rarely  sometimes  often  very often |
| --- | --- | --- |
| 1. | I was short of breath with minimal excursion. | 0 1 2 3 4 |
| 2. | I felt weak or slow. | 0 1 2 3 4 |
| 3. | I was irritable and dissatisfied. | 0 1 2 3 4 |
| 4. | I felt anxious while I was in a crowd (of people). | 0 1 2 3 4 |
| 5. | I felt palpitations. | 0 1 2 3 4 |
| 6. | I had trouble making decisions. | 0 1 2 3 4 |
| 7. | I could not enjoy anything at all. | 0 1 2 3 4 |
| 8. | I did not dare to cross open spaces, such as a public square. | 0 1 2 3 4 |
| 9. | I felt stressed at my work or study. | 0 1 2 3 4 |
| 10. | I argued with others. | 0 1 2 3 4 |
| 11. | I felt chest pain (or pressure). | 0 1 2 3 4 |
| 12. | I looked forward to things. | 0 1 2 3 4 |
| 13. | I thought about my death or suicide. | 0 1 2 3 4 |
| 14. | I did not dare to travel on my own using public transport. | 0 1 2 3 4 |
| 15. | I was dissatisfied with my work or study. | 0 1 2 3 4 |
| 16. | I was hot-tempered without good reason. | 0 1 2 3 4 |
| 17. | I felt dizzy or lightheaded. | 0 1 2 3 4 |
| 18. | I felt like doing things. | 0 1 2 3 4 |
| 19. | I did not want to live anymore. | 0 1 2 3 4 |
| 20. | I had the feeling that I have been working or studying very hard. | 0 1 2 3 4 |
| 21. | I had trouble with controlling my anger. | 0 1 2 3 4 |

| How much trouble did you have: | | never  rarely  sometimes  often  very often |
| --- | --- | --- |
| 22. | I felt a tingling, for example, in my hands. | 0 1 2 3 4 |
| 23. | I could hardly express myself. | 0 1 2 3 4 |
| 24. | I was afraid or anxious. | 0 1 2 3 4 |
| 25. | I did not dare to go alone to a crowded shop. | 0 1 2 3 4 |
| 26. | I was shaking or trembling. | 0 1 2 3 4 |
| 27. | I was afraid of rejection by others. | 0 1 2 3 4 |
| 28. | I was scared. | 0 1 2 3 4 |
| 29. | I was optimistic about my future. | 0 1 2 3 4 |
| 30. | I worked or studied less intensely than before. | 0 1 2 3 4 |
| 31. | I felt shaky or I had shivers. | 0 1 2 3 4 |
| 32. | I felt low and less than others. | 0 1 2 3 4 |
| 33. | I felt jittery and nervous. | 0 1 2 3 4 |
| 34. | I looked forward to my plans and goals for the future. | 0 1 2 3 4 |
| 35. | I had the feeling that I did not do well with my work or study. | 0 1 2 3 4 |
| 36. | I felt uncomfortable when other people looked at me. | 0 1 2 3 4 |
| 37. | I took interest in things. | 0 1 2 3 4 |
| 38. | I felt hopeless. | 0 1 2 3 4 |
| 39. | I was forgetful. | 0 1 2 3 4 |
| 40. | I felt down or depressed. | 0 1 2 3 4 |
| 41. | I felt restless. | 0 1 2 3 4 |
| 42. | I felt energetic and high-spirited. | 0 1 2 3 4 |
| 43. | I wanted to hit people if I was provoked. | 0 1 2 3 4 |
| 44. | I struggled to get the day started. | 0 1 2 3 4 |
| 45. | I felt insecure in the company of others. | 0 1 2 3 4 |
| 46. | I felt tense. | 0 1 2 3 4 |
| 47. | I could not concentrate well. | 0 1 2 3 4 |
| 48. | I worried. | 0 1 2 3 4 |

**SQ-48**

***Scoring of SQ-48:***

All 48 items are scored 0-4 (higher score implies more psychological distress/psychopathology). The following items (VITA) should be rescored: 12, 18, 29, 34, 37, 42. For the subscale WORK, there is also the response “not applicable” for patients/clients who do not work.

*The 48 items are distributed over 9 subscales (7 psychopathology subscales (AGGR, AGOR, ANXI, COGN, MOOD, SOMA, SOPH) and 2 additional subscales (VITA, WORK)):*

AGGR (aggression/hostility 4 items): 10, 16, 21, 43.

AGOR (agoraphobia 4 items): 4, 8, 14, 25

ANXI (anxiety 6 items): 24, 28, 33, 41, 46, 48

COGN (cognitive complaints 5 items): 2, 6, 39, 44, 47

MOOD (depression 6 items): 3, 7, 13, 19, 38, 40

SOMA (somatic complaints 7 items): 1, 5, 11, 17, 22, 26, 31

SOPH (social phobia 5 items): 23, 27, 32, 36, 45

VITA (vitality/optimism 6 items): 12, 18, 29, 34, 37, 42

WORK (work/study functioning 5 items): 9, 15, 20, 30, 35

| *The overall or total score is only based on the 7 psychopathology subscales* | The total score is the sum of all item scores, with the exception of the item scores for WORK and VITA. So, the total score includes 37 items (7 psychopathology subscales): 1, 2, 3, 4, 5, 6, 7, 8, 10, 11, 13, 14, 16, 17, 19, 21, 22, 23, 24, 25, 26, 27, 28, 31, 32, 33, 36, 38, 39, 40, 41, 43, 44, 45, 46, 47, 48. |
| --- | --- |

***References SQ-48:***

Carlier, I.V.E. et al. (2012). Development and validation of the 48 item Symptom Questionnaire (SQ-48) in patients with depressive-, anxiety- and somatoform disorders. *Psychiatry Research*, 200 (2-3), 904-910.

Carlier I.V.E. et al. (2017). Evaluating the responsiveness to therapeutic change with Routine Outcome Monitoring: A comparison of the Symptom Questionnaire-48 (SQ-48) with the Brief Symptom Inventory (BSI) and the Outcome Questionnaire-45 (OQ-45*). Clinical Psychology and Psychotherapy,* 24(1), 61-71.

© ***Copyright SQ-48:***

The Leiden University Medical Centre (LUMC), Department of Psychiatry, Leiden, The Netherlands. Contact person for SQ-48 at the LUMC: I.V.E. Carlier (Email: [I.V.E.Carlier@lumc.nl](mailto:I.V.E.Carlier@lumc.nl)).

| Table 1 Supplementary material | |  |  |  |  |  |  |  |
| --- | --- | --- | --- | --- | --- | --- | --- | --- |
| *Correlation matrix for latent factors in the configural invariance model* | | | | |  |  |  |  |
|  | **Factor** | Agression | Agoraphobia | Anxiety | Cognitive problems | Mood | Somatic Complaints | Socialphobia |
| **Pre-treatment** | Agression | 1.000 |  |  |  |  |  |  |
|  | Agoraphobia | 0.099 | 1.000 |  |  |  |  |  |
|  | Anxiety | 0.245 | 0.627 | 1.000 |  |  |  |  |
|  | Cognitive problems | 0.378 | 0.566 | 0.816 | 1.000 |  |  |  |
|  | Mood | 0.331 | 0.548 | 0.852 | 0.785 | 1.000 |  |  |
|  | Somatic complaints | 0.380 | 0.696 | 0.689 | 0.781 | 0.608 | 1.000 |  |
|  | Socialphobia | 0.415 | 0.367 | 0.632 | 0.800 | 0.669 | 0.598 | 1.000 |
| **Post-treatment** | Agression | 1.000 |  |  |  |  |  |  |
|  | Agoraphobia | 0.190 | 1.000 |  |  |  |  |  |
|  | Anxiety | 0.325 | 0.820 | 1.000 |  |  |  |  |
|  | Cognitive problems | 0.377 | 0.807 | 0.902 | 1.000 |  |  |  |
|  | Mood | 0.226 | 0.643 | 0.760 | 0.730 | 1.000 |  |  |
|  | Somatic complaints | 0.508 | 0.804 | 0.806 | 0.864 | 0.660 | 1.000 |  |
|  | Socialphobia | 0.523 | 0.705 | 0.813 | 0.914 | 0.688 | 0.805 | 1.000 |
| *Note.* Correlations for all factors (sub-scales). All correlations were statistically significant (*p* <0.001). | | | | | | | | |


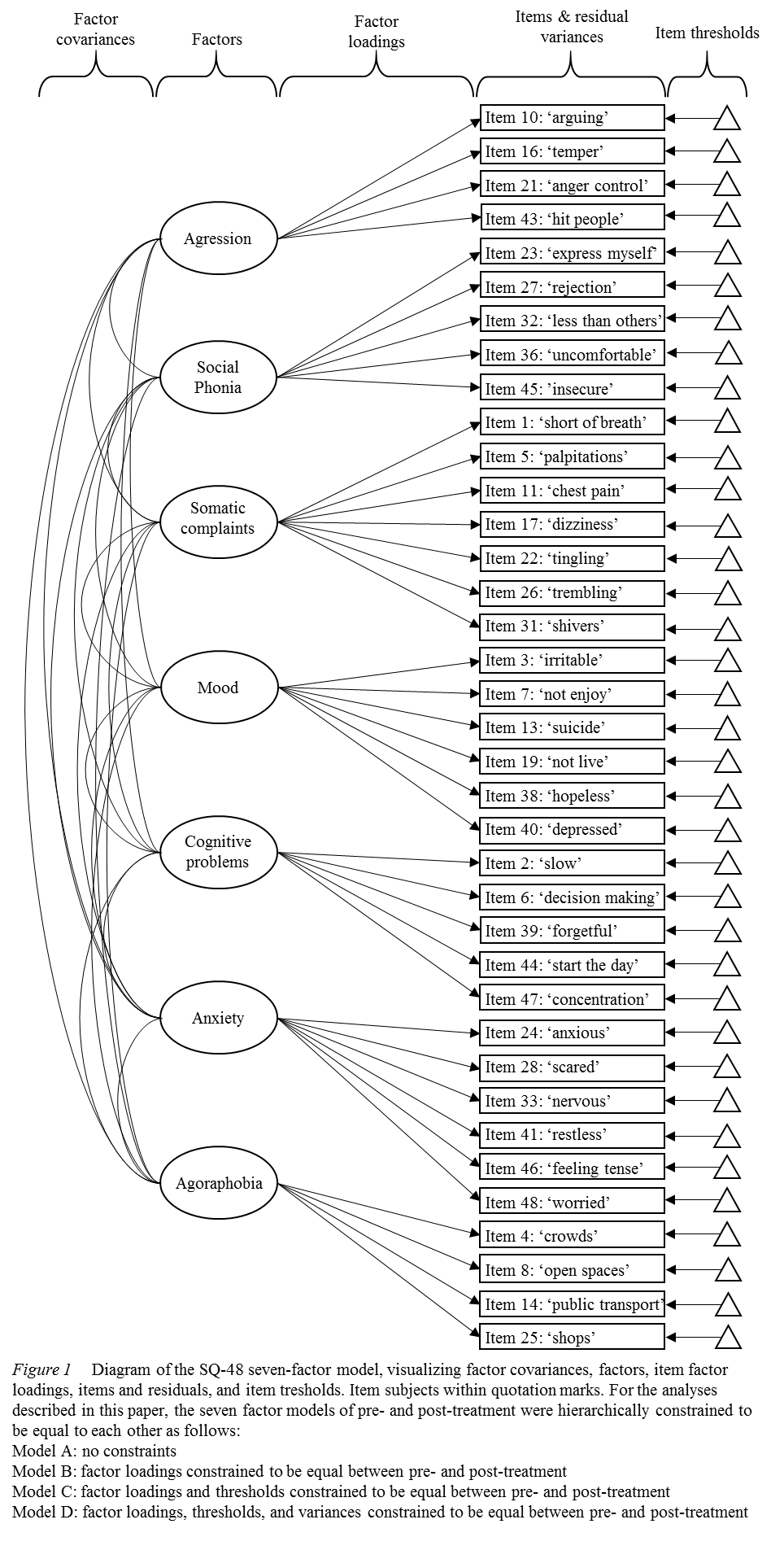


| Table 2 Supplementary material | | |  |  |  |  |  |  |  |  |  |  |  |
| --- | --- | --- | --- | --- | --- | --- | --- | --- | --- | --- | --- | --- | --- |
| *Standardized parameter estimates for the partial metric invariance model of the longitudinal analysis* | | | | | | | |  |  |  |  |  |  |
|  |  | Loadings | | Time 1 | | | | Time 2 | | | | Residuals | |
| Factor | Item | T1 | T2 | Threshold 1 | Threshold 2 | Threshold 3 | Threshold 4 | Threshold 1 | Threshold 2 | Threshold 3 | Threshold 4 | T1 | T2 |
| Agression | 10 | 0.73 (0.04) |  | -0.47 (0.09) | -0.01 (0.09) | 0.67 (0.10) | 1.19 (0.11) | -0.34 (0.09) | 0.21 (0.09) | 0.78 (0.10) | 1.33 (0.12) | - | 0.50 (0.08) |
|  | 16 | 0.96 (0.05) |  | -0.28 (0.08) | 0.26 (0.09) | 0.73 (0.10) | 1.42 (0.13) | -0.04 (0.09) | 0.45 (0.09) | 1.03 (0.11) | 1.71 (0.15) | - | 0.01 (0.14) |
|  | 21 | 0.59 (0.05) |  | -0.12 (0.09) | 0.37 (0.09) | 1.06 (0.11) | 1.66 (0.15) | 0.04 (0.09) | 0.51 (0.09) | 1.19 (0.11) | 1.66 (0.15) | - | 0.65 (0.08) |
|  | 43 | -0.14 (0.08) |  | -0.41 (0.09) | 0.37 (0.09) | 1.24 (0.12) | 2.34 (0.26) | -0.45 (0.09) | 0.26 (0.09) | 1.03 (0.11) | 1.61 (0.14) | - | 0.98 (0.04) |
| Agoraphobia | 4 | 0.87 (0.02) |  | -1.19 (0.11) | -0.76 (0.10) | 0.05 (0.09) | 1.24 (0.12) | -0.94 (0.10) | -0.43 (0.09) | 0.39 (0.09) | 1.22 (0.12) | - | 0.18 (0.05) |
|  | 8 | 0.80 (0.02) |  | -0.49 (0.09) | -0.13 (0.09) | 0.34 (0.09) | 1.22 (0.12) | -0.30 (0.09) | 0.15 (0.09) | 0.68 (0.10) | 1.36 (0.12) | - | 0.31 (0.05) |
|  | 14 | 0.74 (0.04) |  | 0.04 (0.09) | 0.45 (0.09) | 1.08 (0.11) | 1.61 (0.14) | 0.20 (0.09) | 0.68 (0.10) | 1.08 (0.11) | 1.46 (0.13) | - | 0.38 (0.07) |
|  | 25 | 0.84 (0.03) |  | -0.48 (0.09) | -0.17 (0.09) | 0.39 (0.09) | 1.10 (0.11) | -0.39 (0.09) | 0.06 (0.09) | 0.70 (0.10) | 1.19 (0.11) | - | 0.30 (0.05) |
| Anxiety | 24 | 0.72 (0.03) |  | -0.94 (0.10) | -0.49 (0.09) | 0.35 (0.09) | 1.17 (0.11) | -0.86 (0.10) | -0.17 (0.09) | 0.65 (0.10) | 1.24 (0.12) | - | 0.45 (0.05) |
|  | 28 | 0.83 (0.02) |  | -0.55 (0.09) | -0.18 (0.09) | 0.51 (0.09) | 1.39 (0.13) | -0.43 (0.09) | 0.06 (0.09) | 0.59 (0.09) | 1.36 (0.12) | - | 0.29 (0.04) |
|  | 33 | 0.81 (0.02) |  | -1.14 (0.11) | -0.65 (0.10) | 0.07 (0.09) | 1.14 (0.11) | -0.75 (0.10) | -0.35 (0.09) | 0.41 (0.09) | 1.22 (0.12) | - | 0.31 (0.04) |
|  | 41 | 0.87 (0.02) |  | -1.61 (0.14) | -1.06 (0.11) | -0.30 (0.09) | 0.73 (0.10) | -1.19 (0.11) | -0.51 90.09) | 0.25 (0.09) | 1.06 (0.11) | - | 0.21 (0.04) |
|  | 46 | 0.87 (0.02) |  | -1.10 (0.11) | -0.75 (0.10) | 0.04 (0.09) | 0.96 (0.10) | -0.86 (0.10) | -0.31 (0.09) | 0.30 (0.09) | 1.12 (0.11) | - | 0.20 (0.03) |
|  | 48 | 0.80 (0.02) |  | -1.89 (0.18) | -1.24 (0.12) | -0.48 (0.09) | 0.56 (0.09) | -1.33 (0.12) | -0.85 (0.10) | -0.05 (0.09) | 0.85 (0.10) | - | 0.32 (0.05) |
| Cognitive | 2 | 0.60 (0.03) |  | -0.52 (0.09) | -0.15 (0.10) | 0.59 (0.09) | 1.83 (0.17) | -0.45 (0.09) | 0.07 (0.09) | 0.94 (0.10) | 1.97 (0.19) | - | 0.58 (0.06) |
| problems | 6 | 0.65 (0.03) |  | -0.75 (0.10) | -0.37 (0.09) | 0.61 (0.09) | 1.39 (0.13) | -0.62 (0.09) | -0.07 (0.09) | 0.71 (0.10) | 1.53 (0.14) | - | 0.50 (0.06) |
|  | 39 | 0.81 (0.02) |  | -1.19 (0.11) | -0.64 (0.09) | 0.13 (0.09) | 0.99 (0.11) | -0.86 (0.10) | -0.39 (0.09) | 0.45 (0.09) | 1.22 (0.12) | - | 0.28 (0.04) |
|  | 44 | 0.50 (0.04) |  | -0.32 (0.09) | 0.02 (0.09) | 0.47 (0.09) | 1.39 (0.13) | -0.21 (0.09) | 0.18 (0.09) | 0.76 (0.10) | 1.36 (0.12) | - | 0.70 (0.06) |
|  | 47 | 0.72 (0.04) | 0.81 (0.03) | -1.83 (0.17) | -1.14 (0.11) | -0.30 (0.09) | 0.68 (0.10) | -1.24 (0.12) | -0.73 (0.10) | 0.16 (0.09) | 1.06 (0.11) | - | 0.30 (0.05) |
| Mood | 3 | 0.80 (0.04) | 0.80 (0.04) | -1.17 (0.11) | -0.71 (0.10) | 0.17 (0.09) | 1.22 (0.12) | -0.96 (0.10) | -0.44 (0.09) | 0.54 (0.09) | 1.57 (0.14) | - | 0.29 (0.07) |
|  | 7 | 0.75 (0.05) | 0.81 (0.04) | -1.49 (0.13) | -0.86 (0.10) | -0.04 (0.09) | 0.94 (0.10) | -1.03 (0.11) | -0.47 (0.09) | 0.39 (0.09) | 1.33 (0.12) | - | 0.27 (0.06) |
|  | 13 | -0.26 (0.05) |  | -0.78 (0.01) | 0.00 (0.10) | 0.83 (0.10) | 1.76 (0.16) | -0.58 (0.09) | -0.06 (0.09) | 0.86 (0.10) | 1.83 (0.17) | - | 0.79 (0.06) |
|  | 19 | -0.31 (0.05) |  | -0.94 (0.10) | -0.09 (0.09) | 0.76 (0.10) | 1.76 (0.16) | -0.73 (0.10) | -0.17 (0.09) | 0.83 (0.10) | 1.66 (0.15) | - | 0.76 (0.05) |
|  | 38 | -0.19 (0.05) |  | -1.39 (0.13) | -0.51 (0.09) | 0.43 (0.09) | 1.53 (0.14) | -1.17 (0.11) | -0.31 (0.09) | 0.56 (0.09) | 1.49 (0.13) | - | 0.88 (0.04) |
|  | 40 | 0.74 (0.05) | 0.83 (0.04) | -1.39 (0.13) | -0.92 (0.10) | -0.05 (0.09) | 0.96 (0.10) | -1.10 (0.11) | -0.59 (0.09) | 0.32 (0.09) | 1.19 (0.11) | - | 0.22 (0.07) |
| Somatic | 1 | 0.69 (0.03) |  | -0.83 (0.10) | -0.22 (0.09) | 0.58 (0.09) | 1.24 (0.12) | -0.67 (0.10) | 0.04 (0.09) | 0.64 (0.09) | 1.39 (0.13) | - | 0.53 (0.06) |
| complaints | 5 | 0.84 (0.02) |  | -0.55 (0.10) | -0.13 (0.09) | 0.65 (0.10) | 1.14 (0.11) | -0.34 (0.09) | 0.15 (0.09) | 0.83 (0.10) | 1.33 (0.12) | - | 0.22 (0.05) |
|  | 11 | 0.58 (0.04) |  | -0.55 (0.09) | 0.01 (0.09) | 0.96 (0.10) | 1.89 (0.18) | -0.25 (0.09) | 0.37 (0.090 | 1.12 (0.11) | 1.83 (0.17) | - | 0.62 (0.06) |
|  | 17 | 0.68 (0.03) |  | -0.52 (0.09) | -0.09 (0.09) | 0.68 (0.10) | 1.46 (0.13) | -0.32 (0.09) | 0.12 (0.09) | 0.92 (0.10) | 1.27 (0.12) | - | 0.48 (0.06) |
|  | 22 | 0.63 (0.03) |  | -0.44 (0.09) | 0.10 (0.09) | 0.88 (0.10) | 1.61 (0.14) | -0.30 (0.09) | 0.28 (0.09) | 0.97 (0.10) | 1.66 (0.15) | - | 0.54 (0.06) |
|  | 26 | 0.67 (0.05) | 0.80 (0.04) | -0.15 (0.09) | 0.21 (0.09) | 0.75 (0.10) | 1.39 (0.13) | 0.06 (0.09) | 0.52 (0.09) | 1.06 (0.11) | 1.57 (0.14) | - | 0.33 (0.06) |
|  | 31 | 0.46 (0.05) |  | -0.07 (0.09) | 0.35 (0.09) | 0.85 (0.10) | 1.39 (0.13) | 0.17 (0.09) | 0.56 (0.09) | 1.17 (0.11) | 1.89 (0.18) | - | 0.78 (0.06) |
| Socialphobia | 23 | 0.71 (0.03) |  | -0.64 (0.09) | -0.18 (0.09) | 0.75 (0.10) | 1.66 (0.15) | -0.36 (0.09) | 0.00 (0.09) | 0.86 (0.10) | 1.66 (0.15) | - | 0.43 (0.05) |
|  | 27 | 0.76 (0.03) |  | -0.47 (0.09) | 0.00 (0.09) | 0.59 (0.10) | 1.39 (0.13) | -0.40 (0.09) | 0.18 (0.09) | 0.76 (0.10) | 1.57 (0.14) | - | 0.37 (0.05) |
|  | 32 | 0.78 (0.02) |  | -0.61 (0.09) | -0.23 (0.09) | 0.59 (0.10) | 1.57 (0.14) | -0.47 (0.09) | 0.04 (0.09) | 0.85 (0.10) | 1.97 (0.20) | - | 0.35 (0.05) |
|  | 36 | 0.50 (0.04) |  | -0.37 (0.09) | -0.15 (0.09) | 0.45 (0.10) | 1.03 (0.11) | -0.23 (0.09) | 0.21 (0.09) | 0.67 (0.10) | 1.39 (0.13) | - | 0.78 (0.06) |
|  | 45 | 0.70 (0.04) |  | -1.46 (0.13) | -0.96 0.10) | -0.21 (0.09) | 0.83 (0.10) | -1.12 (0.11) | -0.61 (0.09) | 0.20 (0.09) | 1.06 (0.11) | - | 0.50 (0.07) |
| *Note.*  Partial metric model, factor loadings constrained to be equal. All constraints are computed with WLSMV estimation and theta parameterization. Parenthetical values are standard errors. Equality constraints on item loadings of item 3, 7, 26, 40, and 47, are lifted. | | | | | | | | | | | | | |
|  |  |  |  |  |  |  |  |  |  |  |  |  |  |

| Table 3 Supplementary material | | |  |  |  |  |  |  |  |  |  |  |  |
| --- | --- | --- | --- | --- | --- | --- | --- | --- | --- | --- | --- | --- | --- |
| *Standardized parameter estimates for the partial strong invariance model of the longitudinal analysis* | | | | | | | | | | | |  |  |
|  |  | Loadings | |  | | | |  | | | | Residuals | |
| Factor | Item | T1 | T2 | Threshold 1 | Threshold 2 | Threshold 3 | Threshold 4 | Threshold 1 | Threshold 2 | Threshold 3 | Threshold 4 | T1 | T2 |
| Agression | 10 | 0.75 (0.06) |  | -0.49 (0.08) | 0.02 (0.08) | 0.66 (0.09) | 1.21 (0.11) |  |  |  |  | - | 0.48 (0.07) |
|  | 16 | 0.90 (0.06) |  | -0.25 (0.08) | 0.23 (0.08) | 0.73 (0.09) | 1.38 (0.12) |  |  |  |  | - | 0.01 (0.12) |
|  | 21 | 0.59 (0.05) |  | -0.11 (0.07) | 0.38 (0.07) | 1.06 (0.10) | 1.59 (0.13) |  |  |  |  | - | 0.65 (0.07) |
|  | 43 | -0.17 (0.08) |  | -0.46 (0.08) | 0.37 (0.07) | 1.28 (0.11) | 2.14 (0.22) |  |  |  |  | - | 0.98 (0.02) |
| Agoraphobia | 4 | 0.84 (0.03) |  | -1.12 (0.09) | -0.67 (0.08) | 0.11 (0.08) | 1.09 (0.10) |  |  |  |  | - | 0.20 (0.04) |
|  | 8 | 0.74 (0.04) |  | -0.46 (0.07) | -0.08 (0.07) | 0.39 (0.08) | 1.12 (0.10) |  |  |  |  | - | 0.31 (0.05) |
|  | 14 | 0.70 (0.06) |  | 0.03 (0.07) | 0.46 (0.08) | 0.94 (0.09) | 1.34 (0.12) |  |  |  |  | - | 0.41 (0.06) |
|  | 25 | 0.86 (0.04) |  | -0.54 (0.08) | -0.15 (0.08) | 0.45 (0.08) | 1.06 (0.10) |  |  |  |  | - | 0.30 (0.05) |
| Anxiety | 24 | 0.69 (0.04) |  | -1.00 (0.08) | -0.45 (0.07) | 0.35 (0.07) | 1.03 (0.09) |  |  |  |  | - | 0.45 (0.05) |
|  | 28 | 0.81 (0.03) |  | -0.64 (0.08) | -0.21 (0.07) | 0.39 (0.08) | 1.20 (0.10) |  |  |  |  | - | 0.30 (0.04) |
|  | 33 | 0.76 (0.03) |  | -1.04 (0.08) | -0.62 (0.08) | 0.09 (0.07) | 0.98 (0.09) |  |  |  |  | - | 0.32 (0.04) |
|  | 41 | 0.84 (0.03) |  | -1.50 (0.10) | -0.89 (0.08) | -0.18 (0.08) | 0.71 (0.08) |  |  |  |  | - | 0.21 (0.04) |
|  | 46 | 0.83 (0.02) |  | -1.10 (0.08) | -0.66 (0.08) | 0.00 (0.07) | 0.84 (0.09) |  |  |  |  | - | 0.21 (0.03) |
|  | 48 | 0.77 (0.03) |  | -1.64 (0.12) | -1.15 (0.09) | -0.40 (0.07) | 0.54 (0.08) |  |  |  |  | - | 0.33 (0.05) |
| Cognitive | 2 | 0.55 (0.05) |  | -0.54 (0.08) | -0.12 (0.07) | 1.68 (0.08) | 1.68 (0.14) |  |  |  |  | - | 0.60 (0.05) |
| problems | 6 | 0.59 (0.04) |  | -0.72 (0.08) | -0.29 (0.07) | 0.51 (0.08) | 1.25 (0.11) |  |  |  |  | - | 0.53 (0.05) |
|  | 39 | 0.79 (0.03) |  | -1.10 (0.09) | -0.61 (0.08) | 0.17 (0.08) | 1.02 (0.10) |  |  |  |  | - | 0.30 (0.04) |
|  | 44 | 0.45 (0.06) |  | -0.37 (0.08) | 0.03 (0.08) | 0.59 (0.09) | 1.39 (0.12) |  |  |  |  | - | 0.72 (0.05) |
|  | 47 | 0.70 (0.04) | 0.51 (0.06) | -1.83 (0.17) | -1.14 (0.11) | -0.30 (0.09) | 0.68 (0.10) | -1.48 (0.13) | -0.97 (0.11) | -0.08 (0.10) | 0.82 (0.12) | - | 0.33 (0.04) |
| Mood | 3 | 0.80 (0.04) | 0.37 (0.07) | -1.17 (0.11) | -0.71 (0.10) | 0.17 (0.09) | 1.22 (0.12) | -0.84 (0.27) | -0.33 (0.28) | 0.65 (0.29) | 1.68 (0.30) | - | 0.35 (0.06) |
|  | 7 | 0.75 (0.05) | 0.44 (0.07) | -1.49 (0.13) | -0.86 (0.10) | -0.04 (0.09) | 0.94 (0.10) | -0.92 (0.30) | -0.35 (0.29) | 0.50 (0.29) | 1.44 (0.30) | - | 0.34 (0.05) |
|  | 13 | -0.23 (0.05) |  | -0.76 (0.10) | -0.07 (0.09) | 0.93 (0.12) | 2.01 (0.19) |  |  |  |  | - | 0.90 (0.04) |
|  | 19 | -0.29 (0.05) |  | -0.90 (0.11) | -0.17 (0.09) | 0.83 (0.12) | 1.79 (0.17) |  |  |  |  | - | 0.88 (0.04) |
|  | 38 | -0.20 (0.05) |  | -1.34 (0.11) | -0.44 (0.08) | 0.51 (0.09) | 1.57 (0.14) |  |  |  |  | - | 0.95 (0.02) |
|  | 40 | 0.74 (0.04) | 0.45 (0.07) | -1.39 (0.13) | -0.92 (0.10) | -0.05 (0.09) | 0.96 (0.10) | -0.98 (0.31) | -0.48 (0.30) | 0.44 (0.30) | 1.31 (0.30) | - | 0.29 (0.05) |
| Somatic | 1 | 0.72 (0.04) |  | -0.81 (0.08) | -0.17 (0.07) | 0.51 (0.07) | 1.19 (1.19) |  |  |  |  | - | 0.54 (0.05) |
| complaints | 5 | 0.78 (0.04) |  | -0.58 (0.09) | -0.11 (0.08) | 0.67 (0.09) | 1.19 (0.11) |  |  |  |  | - | 0.24 (0.04) |
|  | 11 | 0.55 (0.05) |  | -0.49 (0.08) | 0.13 (0.07) | 1.01 (0.10) | 1.85 (0.15) |  |  |  |  | - | 0.64 (0.05) |
|  | 17 | 0.65 (0.04) |  | -0.53 (0.09) | -0.07 (0.08) | 0.74 (0.09) | 1.29 (0.11) |  |  |  |  | - | 0.50 (0.05) |
|  | 22 | 0.58 (0.05) |  | -0.48 (0.08) | 0.12 (0.08) | 0.90 (0.09) | 1.66 (0.13) |  |  |  |  | - | 0.55 (0.05) |
|  | 26 | 0.67 (0.05) | 0.55 (0.07) | -0.15 (0.09) | 0.21 (0.09) | 0.75 (0.10) | 1.39 (0.13) | -0.14 (0.10) | 0.32 (0.10) | 0.85 (0.12) | 1.37 (0.15) | - | 0.35 (0.05) |
|  | 31 | 0.44 (0.06) |  | -0.01 (0.07) | 0.41 (0.08) | 0.98 (0.11) | 1.62 0.173 |  |  |  |  | - | 0.76 (0.05) |
| Socialphobia | 23 | 0.65 (0.05) |  | -0.57 (0.07) | -0.19 (0.07) | 0.64 (0.08) | 1.43 (0.12) |  |  |  |  | - | 0.45 (0.05) |
|  | 27 | 0.71 (0.04) |  | -0.53 (0.07) | -0.03 (0.07) | 0.53 (0.08) | 1.29 (0.10) |  |  |  |  | - | 0.38 (0.04) |
|  | 32 | 0.74 (0.04) |  | -0.64 (0.08) | -0.21 (0.07) | 0.57 (0.08) | 1.58 (0.12) |  |  |  |  | - | 0.35 (0.05) |
|  | 36 | 0.54 (0.06) |  | -0.40 (0.08) | -0.05 (0.07) | 0.50 (0.08) | 1.16 (0.11) |  |  |  |  | - | 0.76 (0.05) |
|  | 45 | 0.71 (0.05) |  | -1.39 (0.11) | -0.89 (0.09) | -0.12 (0.07) | 0.83 (0.09) |  |  |  |  | - | 0.51 (0.06) |
| *Note.*  Partial strong model, variable thresholds and factor loadings constrained to be equal. All constraints are computed with WLSMV estimation and theta parameterization. Parenthetical values are standard errors. Equality constraints on item loadings and thresholds of item 3, 7, 26, 40, and 47, are lifted.. | | | | | | | | | | | | | |
|  |  |  |  |  |  |  |  |  |  |  |  |  |  |

| Table 4 Supplementary material | | |  | |  | |  | |  | |  | |  | |  | |  | |  | |  | |  | |  |
| --- | --- | --- | --- | --- | --- | --- | --- | --- | --- | --- | --- | --- | --- | --- | --- | --- | --- | --- | --- | --- | --- | --- | --- | --- | --- |
| *Parameter Estimates for the partial strict invariance model of the longitudinal analysis* | | | | | | | | | | | | | | | | | | |  | |  | |  | |  |
|  |  | Loadings | | | |  | | | | | | | |  | | | | | | | | Residuals | | | |
| Factor | Item | T1 | | T2 | | Threshold1 | | Threshold 2 | | Threshold 3 | | Threshold 4 | | Threshold 1 | | Threshold 2 | | Threshold 3 | | Threshold 4 | | T1 | | T2 | |
| Agression | 10 | 0.73 (0.04) | |  | | -0.49 (0.08) | | 0.01 (0.07) | | 0.63 (0.08) | | 1.17 (0.09) | |  | |  | |  | |  | | - | | - | |
|  | 16 | 0.96 (0.05) | |  | | -0.28 (0.08) | | 0.24 (0.08) | | 0.76 (0.09) | | 1.44 (0.11) | |  | |  | |  | |  | | - | | - | |
|  | 21 | 0.59 (0.05) | |  | | -0.12 (0.07) | | 0.37 (0.07) | | 1.05 (0.09) | | 1.59 (0.11) | |  | |  | |  | |  | | - | | - | |
|  | 43 | -0.14 (0.08) | |  | | -0.42 (0.07) | | 0.33 (0.07) | | 1.15 (0.08) | | 1.81 (0.13) | |  | |  | |  | |  | | - | | - | |
| Agoraphobia | 4 | 0.87 (0.02) | |  | | -1.17 (0.09) | | -0.70 (0.08) | | 0.11 (0.08) | | 1.12 (0.09) | |  | |  | |  | |  | | - | | - | |
|  | 8 | 0.80 (0.02) | |  | | -0.50 (0.08) | | -0.09 (0.07) | | 0.40 (0.08) | | 1.19 (0.09) | |  | |  | |  | |  | | - | | - | |
|  | 14 | 0.74 (0.04) | |  | | 0.02 (0.07) | | 0.47 (0.08) | | 0.98 (0.08) | | 1.43 (0.10) | |  | |  | |  | |  | | - | | - | |
|  | 25 | 0.84 (0.03) | |  | | -0.54 (0.08) | | -0.16 (0.08) | | 0.43 (0.08) | | 1.04 (0.09) | |  | |  | |  | |  | | - | | - | |
| Anxiety | 24 | 0.72 (0.03) | |  | | -1.03 (0.08) | | -0.46 (0.07) | | 0.37 (0.07) | | 1.08 (0.09) | |  | |  | |  | |  | | - | | - | |
|  | 28 | 0.83 (0.02) | |  | | -0.64 (0.08) | | -0.21 (0.07) | | 0.40 (0.08) | | 1.22 (0.10) | |  | |  | |  | |  | | - | | - | |
|  | 33 | 0.81 (0.02) | |  | | -1.08 (0.08) | | -0.64 (0.08) | | 0.10 (0.07) | | 1.04 (0.09) | |  | |  | |  | |  | | - | | - | |
|  | 41 | 0.87 (0.02) | |  | | -1.55 (0.10) | | -0.92 (0.08) | | -0.18 (0.08) | | 0.74 (0.08) | |  | |  | |  | |  | | - | | - | |
|  | 46 | 0.87 (0.02) | |  | | -1.14 (0.09) | | -0.68 (0.08) | | 0.01 (0.07) | | 0.89 (0.09) | |  | |  | |  | |  | | - | | - | |
|  | 48 | 0.80 (0.02) | |  | | -1.71 (0.11) | | -1.18 (0.09) | | -0.41 (0.07) | | 0.56 (0.08) | |  | |  | |  | |  | | - | | - | |
| Cognitive | 2 | 0.60 (0.03) | |  | | -0.58 (0.07) | | -0.13 (0.07) | | 0.66 (0.08) | | 1.81 (0.13) | |  | |  | |  | |  | | - | | - | |
| problems | 6 | 0.65 (0.03) | |  | | -0.79 (0.08) | | -0.33 (0.07) | | 0.56 (0.07) | | 1.36 (0.10) | |  | |  | |  | |  | | - | | - | |
|  | 39 | 0.81 (0.02) | |  | | -1.15 (0.09) | | -0.64 (0.08) | | 0.16 (0.08) | | 0.98 (0.09) | |  | |  | |  | |  | | - | | - | |
|  | 44 | 0.50 (0.04) | |  | | -0.35 (0.07) | | 0.03 (0.07) | | 0.53 (0.07) | | 1.29 (0.09) | |  | |  | |  | |  | | - | | - | |
|  | 47 | 0.72 (0.04) | | 0.48 (0.06) | | -1.83 (0.17) | | -1.14 (0.11) | | -0.30 (0.09) | | 0.68 (0.10) | | -1.50 (0.13) | | -0.99 (0.11) | | -0.10 (0.10) | | 0.80 (0.11) | | 0.48 (0.06) | | 0.35 (0.04) | |
| Mood | 3 | 0.80 (0.04) | | 0.36 (0.07) | | -1.17 (0.11) | | -0.71 (0.10) | | 0.17 (0.09) | | 1.22 (0.12) | | -0.75 (0.27) | | -0.24 (0.28) | | 0.74 (0.29) | | 1.77 (0.30) | | 0.36 (0.07) | | 0.37 (0.06) | |
|  | 7 | 0.75 (0.05) | | 0.43 (0.07) | | -1.49 (0.13) | | -0.86 (0.10) | | -0.04 (0.09) | | 0.94 (0.10) | | -0.83 (0.30) | | -0.26 (0.29) | | 0.59 (0.29) | | 1.53 (0.30) | | 0.43 (0.07) | | 0.35 (0.06) | |
|  | 13 | -0.25 (0.05) | |  | | -0.71 (0.08) | | -0.06 (0.08) | | 0.81 (0.08) | | 1.76 (0.12) | |  | |  | |  | |  | | - | | - | |
|  | 19 | -0.30 (0.05) | |  | | -0.87 (0.09) | | -0.17 (0.08) | | 0.76 (0.08) | | 1.67 (0.12) | |  | |  | |  | |  | | - | | - | |
|  | 38 | -0.19 (0.05) | |  | | -1.29 (0.09) | | -0.43 (0.07) | | 0.47 (0.07) | | 1.48 (0.10) | |  | |  | |  | |  | | - | | - | |
|  | 40 | 0.74 (0.05) | | 0.45 (0.07) | | -1.39 (0.13) | | -0.92 (0.10) | | -0.05 (0.09) | | 0.96 (0.10) | | -0.89 (0.31) | | -0.38 (0.30) | | 0.53 (0.30) | | 1.41 (0.30) | | 0.45 (0.07) | | 0.30 (0.06) | |
| Somatic | 1 | 0.69 (0.03) | |  | | -0.85 (0.08) | | -0.19 (0.07) | | 0.51 (0.07) | | 1.22 (0.09) | |  | |  | |  | |  | | - | | - | |
| complaints | 5 | 0.84 (0.02) | |  | | -0.57 (0.08) | | -0.12 (0.07) | | 0.62 (0.08) | | 1.12 (0.09) | |  | |  | |  | |  | | - | | - | |
|  | 11 | 0.58 (0.04) | |  | | -0.48 (0.07) | | 0.11 (0.07) | | 0.95 (0.08) | | 1.77 (0.13) | |  | |  | |  | |  | | - | | - | |
|  | 17 | 0.68 (0.03) | |  | | -0.52 (0.07) | | -0.08 (0.07) | | 0.70 (0.08) | | 1.25 (0.09) | |  | |  | |  | |  | | - | | - | |
|  | 22 | 0.63 (0.03) | |  | | -0.46 (0.07) | | 0.10 (0.07) | | 0.84 (0.08) | | 1.55 (0.11) | |  | |  | |  | |  | | - | | - | |
|  | 26 | 0.67 (0.05) | | 0.55 (0.07) | | -0.15 (0.09) | | 0.21 (0.09) | | 0.75 (0.10) | | 1.39 (0.13) | | -0.17 (0.10) | | 0.29 (0.10) | | 0.82 (0.12) | | 1.34 (0.15) | | 0.55 (0.07) | | 0.36 (0.06) | |
|  | 31 | 0.47 (0.05) | |  | | -0.02 (0.07) | | 0.39 (0.07) | | 0.93 (0.08) | | 1.51 (0.11) | |  | |  | |  | |  | | - | | - | |
| Socialphobia | 23 | 0.71 (0.03) | |  | | -0.61 (0.07) | | -0.21 (0.07) | | 0.69 (0.08) | | 1.55 (0.11) | |  | |  | |  | |  | | - | | - | |
|  | 27 | 0.76 (0.03) | |  | | -0.56 (0.08) | | -0.03 (0.07) | | 0.56 (0.08) | | 1.36 (0.10) | |  | |  | |  | |  | | - | | - | |
|  | 32 | 0.78 (0.02) | |  | | -0.66 (0.08) | | -0.22 (0.07) | | 0.59 (0.08) | | 1.62 (0.12) | |  | |  | |  | |  | | - | | - | |
|  | 36 | 0.50 (0.04) | |  | | -0.38 (0.07) | | -0.05 (0.07) | | 0.48 (0.07) | | 1.12 (0.09) | |  | |  | |  | |  | | - | | - | |
|  | 45 | 0.70 (0.04) | |  | | -1.39 (0.09) | | -0.89 (0.08) | | -0.12 (0.07) | | 0.83 (0.08) | |  | |  | |  | |  | | - | | - | |
| *Note.*  Partial strict model, residual variances, variable thresholds and factor loadings constrained to be equal. All constraints are computed with WLSMV estimation and theta parameterization; residuals were set to 1. Parenthetical values are standard errors.Equality constraints on item loadings, thresholds and risiduals of item 3, 7, 26, 40, and 47, are lifted. | | | | | | | | | | | | | | | | | | | | | | | | |  |
|  |  |  |  |  |  |  |  |  |  |  |  |  |  |  |  |  |  |  |  |  |  |  |  |  |  |

| Table 5 treatment shorter than 16 weeks (median) | | | | | | | | |  |
| --- | --- | --- | --- | --- | --- | --- | --- | --- | --- |
| *Comparison of pre- and post-treatment partial equality constraints concerning SQ-48 sub scales using partial measurement invariance procedures (N=90)* | | | | | | | | | |
| **Models** | **Fit indices** | **Mood** | **Anxiety** | **Somatic complaints** | **Socialphobia** | **Agoraphobia** | **Agression** | **Cognitive problems** | **All factors** |
| **Configural (Model A)** | **χ²** | 230.09 | 38.79 | 103.80 | 18.74 | 2.95 | 2.83 | 20.77 | 3399.76 |
|  | **df** | 18 | 18 | 28 | 10 | 4 | 4 | 10 | 1216 |
|  | **CFI** | 0.832 | 0.994 | 0.947 | 0.989 | >0.999 | >0.999 | 0.984 | 0.922 |
|  | **RMSEA** | 0.364 | 0.114 | 0.174 | 0.099 | <0.001 | <0.001 | 0.110 | 0.142 |
| **Metric (Model B)** | **χ²** | 284.30 | 45.24 | 128.08 | 24.67 | 17.26 | 11.55 | 27.46 | 3601.20 |
|  | **df** | 24 | 24 | 35 | 15 | 8 | 8 | 15 | 1253 |
|  | **CFI** | 0.832 | 0.994 | 0.935 | 0.988 | 0.994 | 0.993 | 0.982 | 0.916 |
|  | **RMSEA** | 0.349 | 0.100 | 0.173 | 0.085 | 0.114 | 0.071 | 0.097 | 0.145 |
| **Partial Metric (Model B)ᵃ** | **χ²** | 239.62 | 45.24 | 118.80 | 24.67 | 17.26 | 11.55 | 27.46 | 3575.686 |
|  | **df** | 20 | 24 | 34 | 15 | 8 | 8 | 15 | 1248 |
|  | ***CFI*** | 0.826 | 0.994 | 0.941 | 0.988 | 0.994 | 0.993 | 0.982 | 0.917 |
|  | **RMSEA** | 0.351 | 0.100 | 0.167 | 0.085 | 0.114 | 0.071 | 0.097 | 0.145 |
| **Partial Strong (Model C)ᵃ** | **χ²** | 240.30 | 50.33 | 124.38 | 33.40 | 17.77 | 10.57 | 30.61 | 3547.29 |
|  | **df** | 25 | 41 | 50 | 29 | 19 | 19 | 29 | 1332 |
|  | ***CFI*** | 0.829 | 0.997 | 0.948 | 0.995 | >0.999 | >0.999 | 0.998 | 0.921 |
|  | **RMSEA** | 0.311 | 0.051 | 0.129 | 0.041 | <0.001 | <0.001 | 0.025 | 0.137 |
| **Partial Strict (Model D)ᵃ** | **χ²** | 249.15 | 55.74 | 136.24 | 34.11 | 23.76 | 17.03 | 35.88 | 3635.82 |
|  | **df** | 28 | 47 | 56 | 34 | 23 | 23 | 34 | 1364 |
|  | ***CFI*** | 0.825 | 0.998 | 0.944 | >0.999 | >0.999 | >0.999 | 0.997 | 0.919 |
|  | **RMSEA** | 0.298 | 0.046 | 0.127 | 0.006 | 0.019 | <0.001 | 0.025 | 0.137 |
|  | **χ²Diff** | 28.55 | 4.28 | 11.98 | 4.15 | 2.95 | 6.45 | 4.65 | 44.32 |
| **A vs. B** | **Δdf** | 6 | 6 | 7 | 5 | 4 | 4 | 5 | 37 |
|  | ***p*** | <0.001 | 0.634 | 0.104 | 0.528 | 0.055 | 0.168 | 0.460 | 0.190 |
|  | **ΔCFI** | 0.038 | >0.001 | 0.012 | 0.001 | 0.006 | 0.007 | 0.002 | 0.006 |
|  | **χ²Diff** | 9.32 | 4.28 | 8.07 | 4.15 | 2.95 | 6.45 | 4.65 | 39.92 |
| **A vs. Bᵃ** | **Δdf** | 2 | 6 | 6 | 5 | 4 | 4 | 5 | 32 |
|  | ***p*** | 0.010 | 0.634 | 0.233 | 0.528 | 0.055 | 0.168 | 0.460 | 0.159 |
|  | **ΔCFI** | 0.006 | >0.001 | 0.006 | 0.001 | 0.006 | 0.007 | 0.002 | 0.005 |
|  | **χ²Diff** | 0.40 | 10.97 | 11.52 | 13.91 | 3.09 | 1.05 | 6.91 | 4.70 |
| **Bᵃ vs. Cᵃ** | **Δdf** | 5 | 17 | 16 | 14 | 11 | 11 | 14 | 84 |
|  | ***p*** | 0.995 | 0.858 | 0.776 | 0.456 | 0.990 | 0.999 | 0.938 | >0.999 |
|  | **ΔCFI** | -0.003 | -0.003 | -0.007 | -0.006 | -0.006 | -0.007 | -0.016 | -0.004 |
|  | **χ²Diff** | 15.75 | 4.87 | 9.98 | 1.400 | 6.23 | 6.51 | 5.86 | 32.77 |
| **Cᵃ vs. Dᵃ** | **Δdf** | 3 | 6 | 6 | 5 | 4 | 4 | 5 | 32 |
|  | ***p*** | 0.002 | 0.561 | 0.126 | 0.924 | 0.183 | 0.164 | 0.320 | 0.429 |
|  | **ΔCFI** | 0.005 | -0.001 | -0.004 | -0.005 | <0.001 | <0.001 | <0.001 | 0.002 |
| *Note.*  Analyses are conducted for all factors (sub scales) combined and separately for the sub scales. A = Configural model, no parameters constrained; B = Partial Metric model, factor loadings are constrained to be equal; Bᵃ = Partial Metric model, invariant factor loadings are constrained to be equal; Cᵇ = Partial Strong model, invariant variable thresholds and factor invariant loadings constrained to be equal; Dᶜ = Partial Strict model, invariant residual variances, invariant variable thresholds, and invariant factor loadings constrained to be equal. All constraints are computed with WLSMV estimation and theta parameterization. SQ-48 = Symptom Questionnaire-48. χ² = Chi-square test df = degrees of freedom for Ch-square test. CFI = comparative Fit Index. χ²Diff = Chi-square difference test. Δdf = degrees of freedom of Chi-square difference test. *p* = p-value*.* ΔCFI = delta comparative fit index. | | | | | | | | | |
| ᵃ Lifting the equality constraints on loadings of items 3, 7, 26, 38, and 40 | | | | | | | | | |

| Table 6 treatment longer than 16 weeks (median) | | | | |  |  |  |  |  |
| --- | --- | --- | --- | --- | --- | --- | --- | --- | --- |
| *Comparison of pre- and post-treatment partial equality constraints concerning SQ-48 sub scales using partial measurement invariance procedures (N=116)* | | | | | | | | | |
| **Models** | **Fit indices** | **Mood** | **Anxiety** | **Somatic complaints** | **Socialphobia** | **Agoraphobia** | **Agression** | **Cognitive problems** | **All long** |
| **Configural (Model A)** | **χ²** | 377.98 | 34.32 | 102.04 | 3.88 | 2.05 | 4.64 | 21.89 | 5107.90 |
|  | **df** | 18 | 18 | 28 | 10 | 4 | 4 | 10 | 1216 |
|  | **CFI** | 0.876 | 0.997 | 0.951 | >0.999 | >0.999 | 0.999 | 0.989 | 0.919 |
|  | **RMSEA** | 0.417 | 0.089 | 0.152 | <0.001 | <0.001 | 0.037 | 0.102 | 0.167 |
| **Metric (Model B)** | **χ²** | 911.99 | 34.32 | 243.77 | 23.05 | 10.81 | 5.91 | 85.72 | 5539.11 |
|  | **df** | 24 | 24 | 35 | 15 | 8 | 8 | 15 | 1253 |
|  | **CFI** | 0.766 | 0.997 | 0.926 | 0.994 | 0.998 | 0.999 | 0.958 | 0.911 |
|  | **RMSEA** | 0.425 | 0.089 | 0.171 | 0.068 | 0.056 | 0.037 | 0.125 | 0.172 |
| **Partial Metric (Model B)ᵃ** | **χ²** | 390.49 | 90.78 | 109.20 | 23.05 | 10.81 | 5.91 | 30.86 | 5452.11 |
|  | **df** | 21 | 24 | 34 | 15 | 8 | 8 | 14 | 1247 |
|  | ***CFI*** | 0.873 | 0.986 | 0.950 | 0.994 | 0.998 | 0.999 | 0.985 | 0.912 |
|  | **RMSEA** | 0.391 | 0.156 | 0.139 | 0.068 | 0.056 | 0.037 | 0.102 | 0.171 |
| **Partial Strong (Model C)ᵃ** | **χ²** | 387.50 | 88.55 | 118.74 | 32.20 | 19.89 | 14.76 | 43.77 | 5339.11 |
|  | **df** | 26 | 41 | 50 | 29 | 19 | 19 | 24 | 1327 |
|  | ***CFI*** | 0.876 | 0.990 | 0.955 | 0.997 | 0.999 | >0.999 | 0.981 | 0.916 |
|  | **RMSEA** | 0.348 | 0.010 | 0.109 | 0.031 | 0.020 | <0.001 | 0.085 | 0.162 |
| **Partial Strict (Model D)ᵃ** | **χ²** | 395.27 | 123.90 | 127.88 | 46.73 | 24.00 | 19.06 | 45.17 | 5545.22 |
|  | **df** | 29 | 47 | 56 | 34 | 23 | 23 | 28 | 1358 |
|  | ***CFI*** | 0.874 | 0.984 | 0.953 | 0.990 | 0.999 | >0.999 | 0.984 | 0.913 |
|  | **RMSEA** | 0.331 | 0.119 | 0.106 | 0.057 | 0.019 | <0.001 | 0.073 | 0.164 |
| **A vs. B** | **χ²Diff** | 248.33 | 23.96 | 63.87 | 11.69 | 6.36 | 1.39 | 38.79 | 69.17 |
|  | **Δdf** | 6 | 6 | 7 | 5 | 4 | 4 | 5 | 37 |
|  | ***p*** | <0.001 | <0.001 | <0.001 | 0.039 | 0.174 | 0.846 | <0.001 | 0.001 |
|  | **ΔCFI** | 0.111 | 0.006 | 0.025 | 0.006 | 0.002 | -0.001 | 0.031 | 0.008 |
| **A vs. Bᵃ** | **χ²Diff** | 9.67 | 23.96 | 4.64 | 11.69 | 6.36 | 1.39 | 6.52 | 55.81 |
|  | **Δdf** | 3 | 6 | 6 | 5 | 4 | 4 | 5 | 31 |
|  | ***p*** | 0.022 | <0.001 | 0.590 | 0.039 | 0.174 | 0.846 | 0.164 | 0.004 |
|  | **ΔCFI** | 0.003 | 0.006 | 0.001 | 0.006 | 0.002 | -0.001 | 0.005 | 0.007 |
| **Bᵃ vs. Cᵃ** | **χ²Diff** | 4.40 | 0.65 | 14.05 | 13.61 | 17.31 | 14.40 | 16.25 | 70.12 |
|  | **Δdf** | 5 | 17 | 16 | 14 | 11 | 11 | 10 | 80 |
|  | ***p*** | >0.999 | >0.999 | 0.595 | 0.480 | 0.099 | 0.211 | 0.093 | >0.999 |
|  | **ΔCFI** | -0.003 | -0.004 | -0.004 | -0.004 | -0.001 | <0.001 | 0.003 | -0.004 |
| **Cᵃ vs. Dᵃ** | **χ²Diff** | 9.65 | 22.56 | 8.15 | 14.25 | 4.84 | 5.08 | 1.80 | 50.64 |
|  | **Δdf** | 3 | 6 | 6 | 5 | 4 | 4 | 4 | 31 |
|  | ***p*** | 0.022 | <0.001 | 0.227 | 0.014 | 0.304 | 0.279 | 0.772 | 0.015 |
|  | **ΔCFI** | 0.002 | 0.006 | -0.002 | 0.007 | <0.001 | <0.001 | -0.002 | 0.004 |
| *Note.*  Analyses are conducted for all factors (sub scales) combined and separately for the sub scales. A = Configural model, no parameters constrained; B = Partial Metric model, factor loadings are constrained to be equal; Bᵃ = Partial Metric model, invariant factor loadings are constrained to be equal; Cᵇ = Partial Strong model, invariant variable thresholds and factor invariant loadings constrained to be equal; Dᶜ = Partial Strict model, invariant residual variances, invariant variable thresholds, and invariant factor loadings constrained to be equal. All constraints are computed with WLSMV estimation and theta parameterization. SQ-48 = Symptom Questionnaire-48. χ² = Chi-square test df = degrees of freedom for Ch-square test. CFI = comparative Fit Index. χ²Diff = Chi-square difference test. Δdf = degrees of freedom of Chi-square difference test. *p* = p-value*.* ΔCFI = delta comparative fit index. | | | | | | | | | |
| ᵃ Lifting the equality constraints on loadings of items 3, 7, 26, 39, and 40 | | | | | | | | | |
